# Serine ADP-ribosylation in *Drosophila* provides insights into the evolution of reversible ADP-ribosylation signalling

**DOI:** 10.1038/s41467-023-38793-y

**Published:** 2023-06-02

**Authors:** Pietro Fontana, Sara C. Buch-Larsen, Osamu Suyari, Rebecca Smith, Marcin J. Suskiewicz, Kira Schützenhofer, Antonio Ariza, Johannes Gregor Matthias Rack, Michael L. Nielsen, Ivan Ahel

**Affiliations:** 1 Sir William Dunn School of Pathology, University of Oxford, South Parks Road, Oxford, OX1 3RE UK; 2 Department of Biological Chemistry and Molecular Pharmacology, Harvard Medical School, Boston, MA USA; 3 Program in Cellular and Molecular Medicine, Boston Children’s Hospital, Boston, MA USA; 4 Proteomics program, Novo Nordisk Foundation Center for Protein Research, Faculty of Health and Medical Sciences, University of Copenhagen, Blegdamsvej 3B, 2200 Copenhagen, Denmark; 5 Present Address: Centre de Biophysique Moléculaire, UPR4301 CNRS, rue Charles Sadron, CEDEX 2, F-45071 Orléans, France; 6 Present Address: School of Biosciences, University of Sheffield, Western Bank, Sheffield, S10 2TN UK; 7 Present Address: MRC Centre for Medical Mycology, School of Biosciences, University of Exeter, Geoffrey Pope Building, Exeter, EX4 4QD UK

**Keywords:** DNA damage response, Mass spectrometry, X-ray crystallography, Evolutionary developmental biology

## Abstract

In the mammalian DNA damage response, ADP-ribosylation signalling is of crucial importance to mark sites of DNA damage as well as recruit and regulate repairs factors. Specifically, the PARP1:HPF1 complex recognises damaged DNA and catalyses the formation of serine-linked ADP-ribosylation marks (mono-Ser-ADPr), which are extended into ADP-ribose polymers (poly-Ser-ADPr) by PARP1 alone. Poly-Ser-ADPr is reversed by PARG, while the terminal mono-Ser-ADPr is removed by ARH3. Despite its significance and apparent evolutionary conservation, little is known about ADP-ribosylation signalling in non-mammalian *Animalia*. The presence of HPF1, but absence of ARH3, in some insect genomes, including *Drosophila* species, raises questions regarding the existence and reversal of serine-ADP-ribosylation in these species. Here we show by quantitative proteomics that Ser-ADPr is the major form of ADP-ribosylation in the DNA damage response of *Drosophila melanogaster* and is dependent on the *d*Parp1:*d*Hpf1 complex. Moreover, our structural and biochemical investigations uncover the mechanism of mono-Ser-ADPr removal by *Drosophila* Parg. Collectively, our data reveal PARP:HPF1-mediated Ser-ADPr as a defining feature of the DDR in *Animalia*. The striking conservation within this kingdom suggests that organisms that carry only a core set of ADP-ribosyl metabolising enzymes, such as *Drosophila*, are valuable model organisms to study the physiological role of Ser-ADPr signalling.

## Abstract (web summary)

In the DNA damage response, ADP-ribosylation is an essential signaling pathway. Here the authors utilize a multidisciplinary approach to establish its molecular basis in fruit flies and provide evidence for Drosophila’s suitability as model organism.

## Introduction

ADP-ribosylation (ADPr) is a post-translational modification of proteins that entails the transfer of ADP-ribose moieties from NAD^+^ onto a target protein. It is involved in the regulation of a diverse range of cellular processes, such as DNA repair, transcriptional regulation, immunity, and microbial metabolism, amongst others^1–4^. ADP-ribose units can be attached to a variety of amino acid side chains amongst others with acidic (Glu/Asp), basic (Arg/Lys), hydroxyl (Ser/Tyr), and thiol (Cys) functionalities^2,5^. Some writers, such as PARP1, PARP2, and tankyrase1/2 (also termed PARP5a/b) can extend the initial modification known as mono(ADP-ribosylation) (MARylation) and create linear or branched ADP-ribose polymers known as poly(ADP-ribosylation) (PARylation)^6–8^.

The binding of PARP1/2 induces PARP1/2-dependent protein ADPr at DNA breaks, which gives rise to ADPr signals that activate and control a variety of DNA damage response (DDR) mechanisms required for the decompaction of chromatin and the recruitment of repair factors^4,9^. Earlier studies showed that PARP1 and PARP2 catalyse glutamate/aspartate modification in vitro^10^, while mass-spectrometric analysis revealed that serine-ADPr is the main residue modified by ADPr during DNA damage in human cells^11–14^. This discrepancy was resolved with the discovery of the auxiliary protein, histone PARylation factor 1 (HPF1)^11,15^, which completes the active site of PARP1/2 by contributing substrate-binding and catalytic residues^16–18^. In addition, the PARP1/2:HPF1 complex is also responsible for the less understood modification of tyrosine residues^19,20^.

ADPr is highly dynamic and must be kept tightly regulated due to the associated high energy expenditure. Therefore, once a suitable cellular response has been achieved, ADPr signalling ceases and the utilised ADP-ribose units are recycled by specialised erasers that convert the ADP-ribose into other nucleotides including ATP and NAD^+^^21^. The main enzyme responsible for degrading the bulk of PAR chains is poly(ADP-ribose)glycohydrolase (PARG), which hydrolyses the acetal bond within the ADP-ribose polymer, but cannot reverse the protein-ribose linkage^22–24^. In human cells, this specific reaction, removal of Ser-ADPr, is carried out by (ADP-ribosyl)hydrolase 3 (ARH3)^23,25^.

Notably, the interplay of ADPr establishment by the PARP1/2:HPF1 complexes with the stepwise modification removal by PARG and ARH3 is integral for the control of DNA repair and chromatin structure regulation^8,15,26–28^. Recently it was also shown that these components are critical determinants of the response to clinically relevant PARP inhibitors^27,29,30^. Despite the importance of Ser-ADPr signalling in mammals, its relevance for *Animalia* outside the mammalian lineage remains elusive. Whilst PARPs and ADPr have previously been studied in *Drosophila melanogaster*^31^, the nature of the residue predominantly modified with ADPr in this model organism has yet to be established. *Drosophila* Parp (*d*Parp) and Parg (*d*Parg) have also been shown to be implicated in several biological functions such as the DDR^32^, transcriptional regulation^33,34^, and chromatin remodelling^35–39^ among others. Furthermore, *d*Parp was found to be an essential gene in *Drosophila*, with deletion being lethal during the transition from the larval to the pupal stage^34,40^. Expression of loss-of-function mutations of d*Parg* induces a lethal larval phenotype at 25 °C, too. However, 25% of mutant flies were able to progress to the adult stage at 29 °C, albeit presenting a progressive neurodegeneration phenotype linked to PAR accumulation in neurons^41^. Moreover, manipulation of d*Parp* or d*Parg* gene expression levels led to altered phenotypes in fly models of neurodegenerative diseases such as Parkinson’s disease^42,43^, Alzheimer’s disease^44^, and amyotrophic lateral sclerosis^45^. However, it is not known if *d*Parp cooperates with *d*Hpf1 and, if so, whether the resultant modification is predominantly localised to serine residues.

Here, we report the existence of an abundant and conserved Ser-ADPr signalling system in *Drosophila* catalysed by *d*Parp:*d*Hpf1 with a functionality largely comparable to the DDR-induced ADPr signalling in humans. We further show that while *Drosophila* lacks ARH3, there is a striking evolutionary adaptation of *d*Parg that confers functional equivalency to both human PARG and ARH3. The conservation and relative simplicity of the Ser-ADPr in *Drosophila* – with only one DNA repair-associated PARP and one opposing hydrolase – makes fruit flies an attractive model for further investigation of this important modification.

## Results

### Serine ADP-ribosylation in *D. melanogaster*

**Fig. 1:**
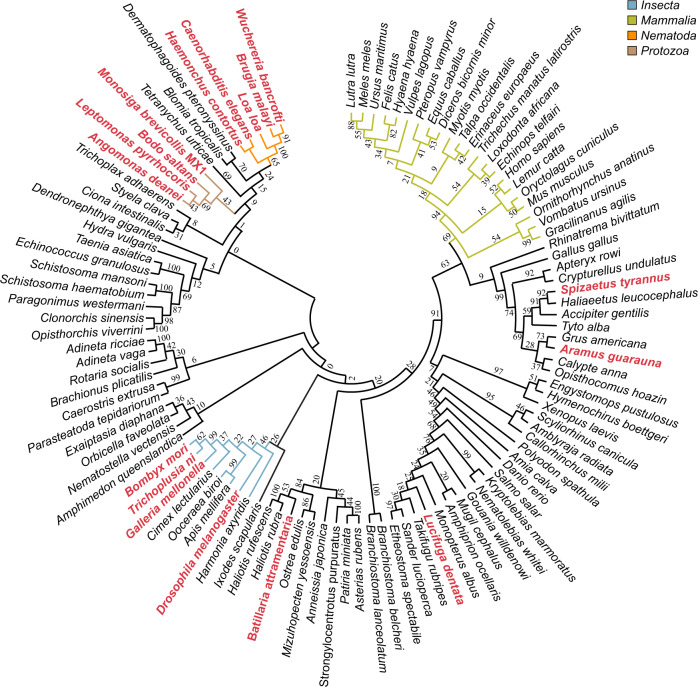
Evolutionary analysis of HPF domain. The phylogenetic tree was constructed with amino acid sequences isolated from their whole protein context by multiple sequence alignment. The evolutionary history was inferred using the maximum-likelihood method under the LG model of amino acid substitution as implemented in MEGA11. The bootstrap consensus tree is shown and was inferred from 1000 replicates to represent the evolutionary history of the taxa analysed. Species in which no ARH3 could be identified by BLAST search are highlighted in red. Relevant clades are highlighted by coloured branches (*Insecta*, blue; *Mammalia*, green; *Nematoda*, orange; *Protozoa*, brown). Sequence data are provided in Supplementary Data 1.

To date, Ser-ADPr has only been studied in vertebrates. Our phylogenetic analysis revealed that HPF1 has been conserved in virtually all *Metazoa*, including primitive branches like corals and sponges as well as organisms known for frequent gene losses such as *D. melanogaster* and *Caenorhabditis elegans* (Fig. 1 and Supplementary Data 1). In addition, HPF1 is found in many *Protozoa*. Similarly, ARH3 is wildly distributed amongst the *Metazoa*, but can only be found sporadically within the *Amoebazoa* and *Alveolata* (Supplementary Data 1). The appearance of these two crucial genes for Ser-ADPr establishment and removal in the early evolution of the *Animalia* kingdom strongly suggests this as the origin of this ADP-ribosylation signalling variation. Surprisingly, our analysis revealed that ARH3 is missing in several eukaryotic lineages including *Nematoda*, *Lepidoptera* and most *Diptera*, including all *Drosophila* species. However, these ARH3-deficient species still retain the main PAR degrader PARG as well as HPF1. These findings suggest that the Ser-ADPr cycle in *Drosophila* may differ from other *Metazoa* and therefore warrants a more detailed investigation.

**Fig. 2:**
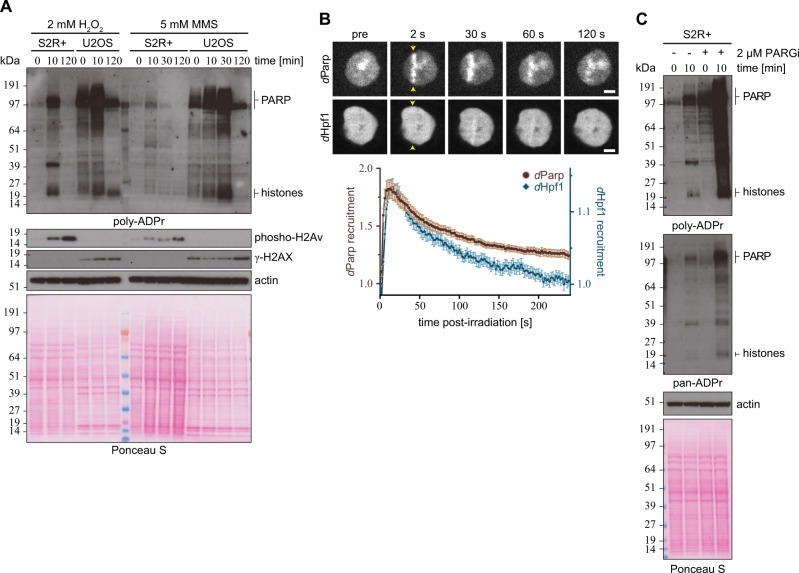
ADP-ribosylation upon genotoxic stress in *Drosophila* S2R+ cells. **A** *Drosophila* S2R+ and human U2OS cells were treated with either 2 mM H_2_O_2_ or 5 mM MMS and analysed at indicated time points. The cells were lysed, and proteins were separated by SDS-PAGE and then analysed for poly-ADPr levels by immunoblot. Actin and Ponceau S staining was used as a loading controls. The ‘PARP’ and ‘histones’ labels next to the image denote the approximate sizes where these proteins can be found. The experiment was repeated independently three times with similar results. **B** Representative images (top) and kinetics (bottom) of EGFP-*d*Parp and EGFP-*d*Hpf1 recruitment to sites of DNA damage induced by 405 nm laser irradiation, in *Drosophila* S2R+ cells. Scale bar, 2 µm. Data from B are a representative of 3 independent replicates (6–10 cells per replica per condition) with *n* = 24 cell for EGFP-*d*Parp and *n* = 22 cell for EGFP-*d*Hpf1 and represent normalised mean values ± SEM. Sites of irradiation are indicated by yellow arrows. **C** S2R+ cells were pre-treated with DMSO or 2 μM PARGi (PDD00017273) for 16 h followed by 2 mM H_2_O_2_ treatment for the indicated time in the absence or presence of PARGi. Poly-ADPr (left panel) and pan-ADPr (right panel) levels were analysed by immunoblot. The ‘PARP’ and ‘histones’ labels next to the image denote the approximate sizes where these proteins can be found. The experiment was repeated independently three times with similar results.

To investigate this hypothesis, we first confirmed that all components – *d*Parp, *d*Hpf1 and *d*Parg –localised to the nucleus in S2R+ cells (Supplementary Fig. 1A) before progressing to elucidate ADPr dynamics in *Drosophila* after DNA damage. We exposed S2R+ cells to DNA damage by hydrogen peroxide (H_2_O_2_) and methyl methanesulfonate (MMS) then compared the ADPr pattern before and after DNA damage by using different anti-ADPr antibodies and reagents (Fig. 2A). We observed that ADPr is swiftly (< 10 min) induced after H_2_O_2_ exposure and decays rapidly post-stress (< 120 min; Fig. 2A). In contrast, the alkylating agent MMS showed a less pronounced ADPr response comparable to U2OS cells under the same assay conditions (Fig. 2A). The overall pattern of poly-ADPr mirrors that of human U2OS cells with prominent bands corresponding to histones and PARP1. While we cannot fully exclude a different origin of the signal, the apparent similarity of the S2R+ ADPr signal with the human U2OS cell, for which histones and *h*PARP1 have been reported as the two major targets of DNA damage-induced ADPr^11,12,15^, strongly suggests this ADPr signal relates to histones and *d*Parp. Furthermore, these data revealed that *Drosophila* and human cells exhibit comparable ADPr dynamics in response to DNA damage^14,15,25^. To determine if *d*Parp and *d*Hpf1 are actively recruited to sites of DNA lesions, S2R+ cells were transfected with GFP-*d*Parp or GFP-*d*Hpf1 and subjected to laser microirradiation coupled to live-cell imaging (Fig. 2B). We observed a robust recruitment of *d*Parp to sites of damage within seconds of laser-induced damage, comparable to what has been described with *h*PARP1^28,46^. Similarly, we also observed the rapid recruitment of *d*Hpf1 to sites of damage, albeit not to the same extent as *d*Parp. This mimics the human recruitment profile where PARP1 exceeds HPF1 recruitment^28^.

Canonical PAR hydrolase activity of *d*Parg has been shown^32,41,47,48^. To confirm that *d*Parg regulates cellular PARylation levels during DNA damage, we treated S2R+ cells with the PARG inhibitor PDD00017273 (PARGi)^49^. When compared to control cells that were treated with dimethyl sulfoxide (DMSO) as a negative control, PARGi-treated S2R+ cells showed higher levels of PARylated proteins in the unstimulated state (Fig. 2C). We also confirmed that PARylation was induced in PARGi-treated S2R+ cells following DNA damage (Fig. 2C). By contrast, we detected only negligible differences in pan-ADPr (combination of both MAR- and PARylation) between DMSO and PARGi-treated S2R+ cells under the unstimulated condition (Fig. 2C). However, we observed that ADPr was dramatically stimulated in PARGi-treated S2R+ cells upon DNA damage in comparison to DMSO-treated S2R+ cells (Fig. 2C). These data suggest that the PARGi developed against human PARG (*h*PARG) efficiently inhibited *d*Parg activity, thereby blocking degradation of ADPr in *Drosophila* and allowing enrichment of ADPr sites.

**Fig. 3:**
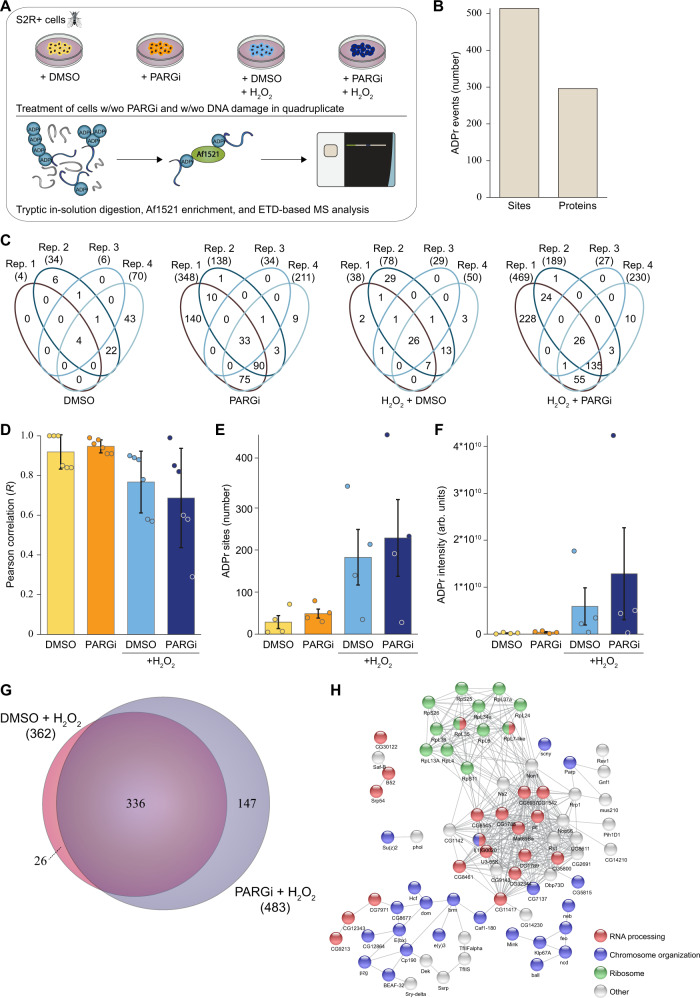
Mass spectrometric identification of ADPr sites in S2R+ cells. **A** Experimental overview. S2R+ cell cultures were treated with DMSO or 2 μM PARGi (PDD00017273) under control conditions or DNA damage conditions (H_2_O_2_) in quadruplicate. Lysates were in-solution digested, and ADPr-modified peptides were enriched using in-house produced GST-tagged Af1521. ADPr samples were analysed on a Thermo Orbitrap Fusion Lumos using EThcD-based fragmentation. The Figure was partly generated using Servier Medical Art, provided by Servier, licensed under a Creative Commons Attribution 3.0 unported license. **B** Histogram showing the total number of identified and localised ADPr-modified sites and proteins. **C** Venn diagrams depicting the distribution of unique ADP-ribosylated peptides identified across the four different approaches. **D** Average Pearson correlation of identified ADPr sites from the four conditions. Represented are mean values of *n* = 6  ±SD. **E** Overview of the number of identified and localised ADPr sites. *n* =  4 cell culture replicates, data are presented as mean values  ±  SEM. (**F**) As (**D**), showing ADPr intensity. Each cell culture condition was prepared in quadruplicates and data are presented as mean values ±  SEM. **G** Scaled Venn diagram depicting the overlap between ADPr sites identified under DMSO and PARGi conditions, both upon H_2_O_2_ treatment. **H** STRING network visualising functional interactions between proteins with ADPr sites specifically found under PARGi-treated conditions. Minimum required interaction score was set to high confidence (0.7), and disconnected proteins were omitted from the network. Proteins were annotated with colours as highlighted in the figure legend.

Next, we aimed to identify the specific proteins that are ADP-ribosylated in *Drosophila* S2R+ cells by mass spectrometric analysis. To this end, we employed the well-established Af1521 enrichment approach^50–52^, and analysed protein extracts from DMSO- and PARGi-treated S2R+ cells in the absence or presence of DNA damage (Fig. 3A). Overall, we confidently identified 514 ADPr sites (localisation probability >0.9), corresponding to 296 ADPr target proteins in *Drosophila* S2R+ cells (Fig. 3B, C and Supplementary Data 2). Reassuringly, the data demonstrated good localisation probability with >75% of the ADPr peptide spectrum matches (PSMs) possessing a localisation probability > 90% (Supplementary Fig. 2A). Overall, we observed a high degree of reproducibility between our experimental replicates, with the most variation present in the H_2_O_2_-treated samples (Fig. 3C, D and Supplementary Fig. 2B).

We identified the highest number of ADPr sites in PARGi samples treated with H_2_O_2_ (483 in total) followed by DMSO samples treated with H_2_O_2_ (362 in total, Supplementary Fig. 2C). Overall, DNA damage resulted in the greatest difference in the ADP-ribosylome (Supplementary Fig. 2D), and on average, we noticed the highest increase in the number of ADPr sites when comparing DMSO-treated cells exposed to DNA damage to no-damage DMSO-treated cells, with ~6 times more sites being detected upon H_2_O_2_ treatment (Fig. 3E). The same trend was observed in PARGi-treated cells, with ~5 times more sites being detected upon H_2_O_2_ treatment. This increase in number of ADPr sites upon DNA damage was even more prominent for the intensity of ADPr-modified peptides (Fig. 3F). Here, we observed on average ~29 times and ~30 times more ADPr intensity for H_2_O_2_-treated DMSO samples and PARGi samples, respectively. For DNA damage-induced samples, the addition of PARGi resulted in ~2 times more intensity compared to the DMSO condition. Upon DNA damage induction, the overlap between DMSO-treated and PARGi-treated cells was high (66%), whereas ~29% of the sites were specific for PARGi-treated samples (Fig. 3G). The latter fraction most likely represents physiologically low abundance or rapid turnover sites that were stabilised by PARGi-treatment. The sites specific for PARGi-treated samples under DNA damage conditions were enriched in proteins involved in RNA processing, chromosome organization and ribosome (Fig. 3H). However, statistically significant changes were limited to five sites upregulated upon PARGi treatment and one site upregulated in DMSO-treated samples (Supplementary Fig. 2E).

**Fig. 4:**
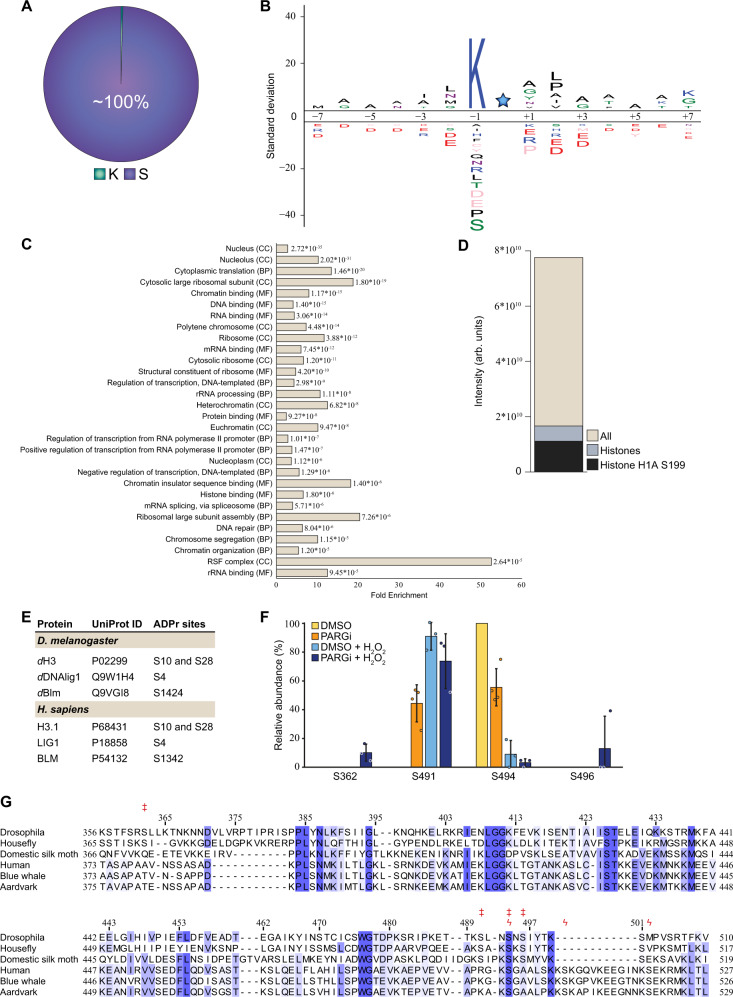
Site-specific properties of the ADP-ribosylome. **A** Pie chart visualising the distribution of ADPr-modified amino acid residues. **B** IceLogo analysis showing the sequence context surrounding identified serine ADPr sites (light blue star), with amino acid residues above the line being enriched. Sequence windows from all serine residues in ADPr target proteins were used as a reference. **C** Gene Ontology analysis visualising the enrichment of all Ser-ADPr target proteins compared to the total genome. CC Cellular compartment; BP Biological process; MF Molecular function. **D** Histogram showing the overall intensity of ADPr sites, the ADPr intensity from histones, and the ADPr intensity from histone H1A Ser199. **E** Table comparing ADPr sites identified in *Drosophila* to ADPr sites identified in human. **F** *d*Parp automodification analysis, showing the relative modification abundance based on MS/MS intensity. *n* = 4 cell culture replicates, data are presented as mean values ± SD. **G** Multiple sequence alignment of selected insect and mammalian PARP automodification domain sequences. Ser-ADPr sites identified in *d*Parp and *h*PARP1 are indicated above the alignment by double-dagger (‡) and koppa (ϟ) symbol, respectively. Indexing indicates *d*Parp residue position. Extended insect alignment is provided in Supplementary Fig. 2 and sequences in Supplementary Table 1.

As observed in human cells^11,12,14,50^, virtually all identified ADPr acceptor sites detected in this study localised to serine residues under these experimental conditions (Fig. 4A and Supplementary Data 2), demonstrating that Ser-ADPr is the main form of ADPr in the *Drosophila* DDR as observed in humans. Given that our previous investigation into Af1521-enrichment approach did not indicate any biased towards a specific protein-ADP-ribose linkages^50^, we suggest that if modification of other amino acid residues occurs in *Drosophila* their abundance might be below the detection limit. We found that most ADP-ribosylated serine residues (69.4%) resided in the lysine-serine (KS) motif as seen in humans^13,51^, suggesting that the targeting consensus of Ser-ADPr is evolutionally conserved (Fig. 4B and Supplementary Data 2). Similarly, the Ser-ADPr targets are primarily nuclear proteins associated with the maintenance of genome stability and chromatin structure (Fig. 4C).

In human cell lines, histones have been shown to be a major target of ADPr^50,53^, and we confirm this to also be the case in *Drosophila* (Fig. 4D). Specifically, we found that *Drosophila* histone H1 is one of the most ADP-ribosylated proteins, with modifications on multiple sites and Ser199 identified as the most abundantly modified residue (Fig. 4D, Supplementary Fig. 3A and Supplementary Data 2). While none of the histone H1 ADPr sites are identical to the sites found on human histone H1, a number of other *Drosophila* Ser-ADPr sites in other proteins are identical to those in their human homologues. For example, here we observed ADPr on Ser10 and Ser28 on *Drosophila* histone H3, which are also modified on human histone H3 in accordance with previous observations^11,12,14,19,50^. Whereas S10 ADPr was observed in *Drosophila* under physiological conditions, ADPr on Ser28 was only observed upon DNA damage. *Drosophila* DNAlig1 was ADP-ribosylated on Ser4, which is also seen in human Lig1^11,12^. Another example was *Drosophila* BLM, which was ADP-ribosylated on Ser1424, corresponding to human Ser1342 (Fig. 4E)^12^.

In addition to these *trans* targets, *d*Parp was auto-ADP-ribosylated on four serine residues (Ser362, Ser491, Ser494, and Ser496) with the intensity of these modification sites following the global trend for total ADPr with regard to PARGi and H_2_O_2_ treatments (Supplementary Fig. 3B–E and Supplementary Data 2). However, the relative fraction of ADPr intensity varied across the different conditions, with Ser494 being most abundantly modified under control conditions, and Ser491 upon DNA damage (Fig. 4F). The observed automodification of *d*Parp was located to a region corresponding to the earlier described automodification domain of human PARP1 (*h*PARP1; aa 373–527), which contains the main acceptor sites Ser499, Ser507, and Ser519^11,30,50^. Among the four *d*Parp automodification sites, Ser491 and Ser496 are absolutely, Ser494 partially (75%, 27 of 36 selected insect species) and Ser362 not conserved in insects (Supplementary Fig. 4 and Supplementary Data 1). Furthermore, the sequence context of the PARP1 automodification sites of mammals and insects is conserved within, but not across, their respective phylogenetic classes. It is striking that the positioning of the main automodification sites relative to the other PARP1 domains/regions is highly conserved (Fig. 4G). This suggests that the potential role of PARP1 automodification in the regulation of its recruitment and release from sites of DNA damage relies on relative positioning within the structural rather than the exact amino acid context. This is further supported by the nature of the PARP1 automodification, which consists of elongated and branched ADP-ribose chains that may prevent recognition of the sequence context.

While most of the identified Ser-ADPr protein targets were shared between human and *Drosophila*, we also identified 25 *Drosophila*-specific Ser-ADPr targets (including eleven proteins of unknown function). The gene ontology analysis shows strong enrichment of cellular components associated with chromosomes such as the heterochromatic region and polytene chromosome (Fig. 4C). For example, ADPr on Ser21 of D1, a multi-AT-hook chromosomal protein, displayed the second highest intensity in total proteins while *Drosophila* HP5, identified as an HP1 interactor, held six Ser-ADPr modification sites (Ser98, Ser101, Ser211, Ser249, Ser347, and Ser399).

**Fig. 5:**
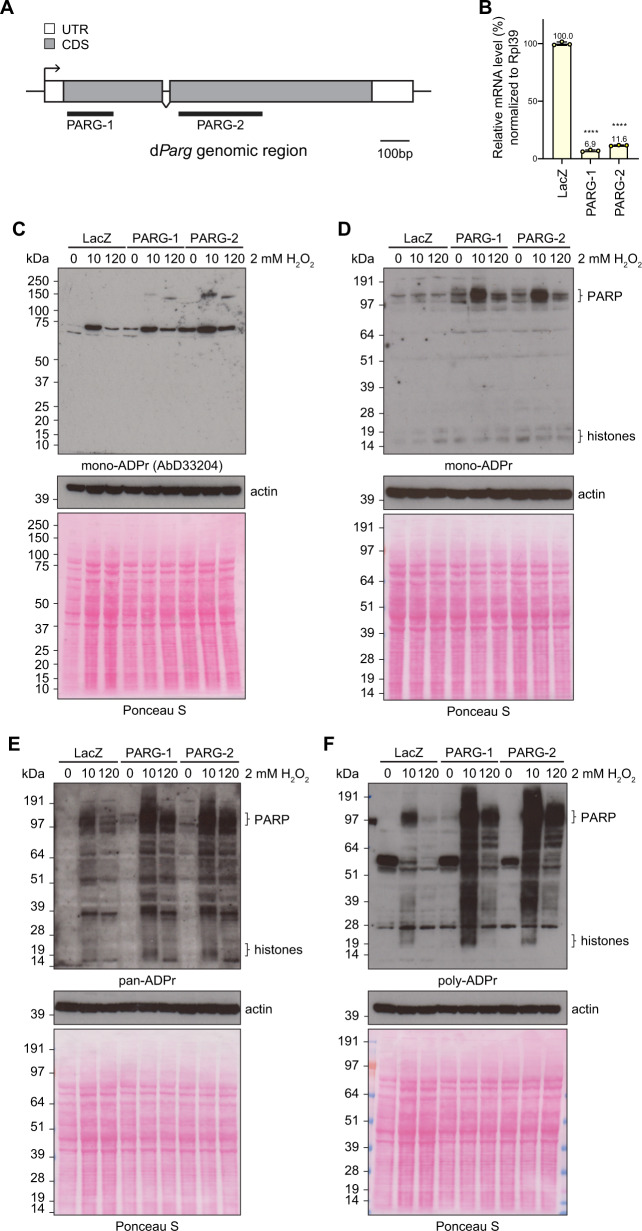
*d*Parg removes mono-Ser-ADPr in vivo. **A** Schematic structure of the d*Parg* gene genomic region. White boxes and grey boxes show the untranslated region and the coding region of the d*Parg* gene, respectively. Black overlines show regions targeted by PARG dsRNAs. **B** The relative gene expression analysis of the d*Parg* gene in S2R+ cells as determined by RT-qPCR and normalised using the *RpL39* gene as an internal control. Error bars indicate average SD from three independent replicas. Asterisks indicate statistical significance compared with the control, as determined by t-test (****p < 0.0001, Two-tailed P-value, PARG-1 vs LacZ, *p* = 6.9 × 10^−8^, PARG-2 vs LacZ, *p* = 6.3 × 10^−8^). *Drosophila* S2R+ cells were treated with 2 mM H_2_O_2_ analysed at indicated time-points. **C**–**F** Proteins from whole-cell lysates were separated by SDS-PAGE and then analysed by immunoblot using mono-ADPr- (MAR antibody AbD33204. **C** mono-ADPr- (MAR detection reagent MABE1076. **D** pan-ADPr- (Both PAR and MAR detection reagent, MABE1016. (**E**) and poly-ADPr- (antibody 4336-BPC-100. **F** Binding reagent. Ponceau S staining and actin were used as a loading control. The ‘PARP’ and ‘histones’ labels next to the image denote the approximate sizes where these proteins can be found. These experiments were repeated independently three times with similar results.

Interestingly, PARGi treatment doubled the abundance of Ser-ADPr sites, indicating that *d*Parg may be responsible for removing mono-Ser-ADPr in *Drosophila* (Fig. 4E). To confirm this, we first established that *d*Parg recruits to sites of laser-induced DNA damage (Supplementary Fig. 1B). We then investigated the ADPr pattern of S2R+ cells subjected to either a double stranded RNA (dsRNA)-mediated knockdown of the *d*Parg gene or the *LacZ* gene (used as a control) before and after exposure to H_2_O_2_ (Fig. 5). Two different dsRNAs, corresponding to different parts of the coding DNA sequences of the *d*Parg genes (Fig. 5A), were used to confirm that the effects were specific to *d*Parg depletion (Fig. 5B). Next, we analysed the products of PARP-mediated ADPr before and after H_2_O_2_ treatment by immunoblot of whole-cell extracts. Interestingly, levels of protein mono-ADPr in both *d*Parg knockdown cell lines before and after DNA damage were increased (Fig. 5C–E), while polymer levels only increased after H_2_O_2_ exposure (Fig. 5E, F). This suggests that *d*Parg can cleave terminal linkages in vivo and, together with our ADP-ribosylomics data, points towards serine residues as targets for reversible DNA damage-induced ADPr.

### *d*Parg can hydrolyse Ser-ADPr in vitro

**Fig. 6:**
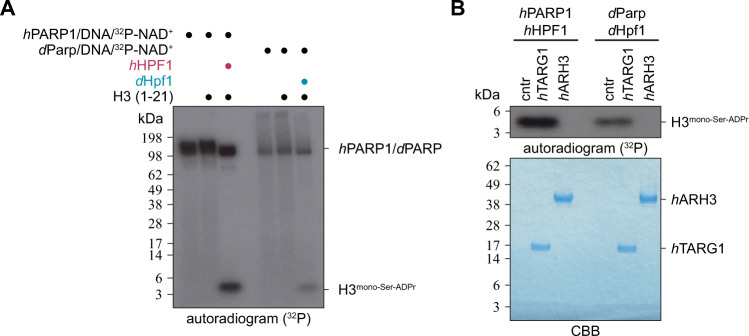
*d*Parp writes serine-ADP-ribosylation in a *d*Hpf1-dependent manner. **A** Mono-Ser-ADPr of H3 histone peptide (aa 1–21) was obtained by incubation with recombinant *h*PARP1:*h*HPF1 complex, or recombinant *d*PARP1:*d*HPF1 complex using ^32^P-NAD^+^ as the ADP-ribose donor. PARP reactions were stopped by the addition of olaparib. The experiment was repeated independently three times with similar results. **B** ADP-ribosylated histone H3 peptides from the (**A**) were purified from the reaction and treated with recombinant *h*ARH3 to confirm the presence of Ser-ADPr. The experiment was repeated independently three times with similar results.

Next, we decided to reconstitute Ser-ADPr in vitro using *Drosophila* proteins. First, we demonstrated that the recombinant *d*Parp1:*d*Hpf1 complex can efficiently ADP-ribosylate the histone H3 tail in vitro, as previously shown for recombinant *h*PARP1:*h*HPF1 (Fig. 6A)^11,15,18^. To confirm the nature of the modification, we purified the modified peptide and incubated it with two human (ADP-ribosyl)hydrolases, *h*TARG1 and *h*ARH3, which are specific for Glu/Asp- and Ser-linked ADPr respectively^21,25,54^. Here we were able to show that *h*ARH3 efficiently removed ADPr from the histone H3 peptide, whereas *h*TARG1 did not, thus strongly suggesting that the modification is indeed Ser-ADPr (Fig. 6B).

**Fig. 7:**
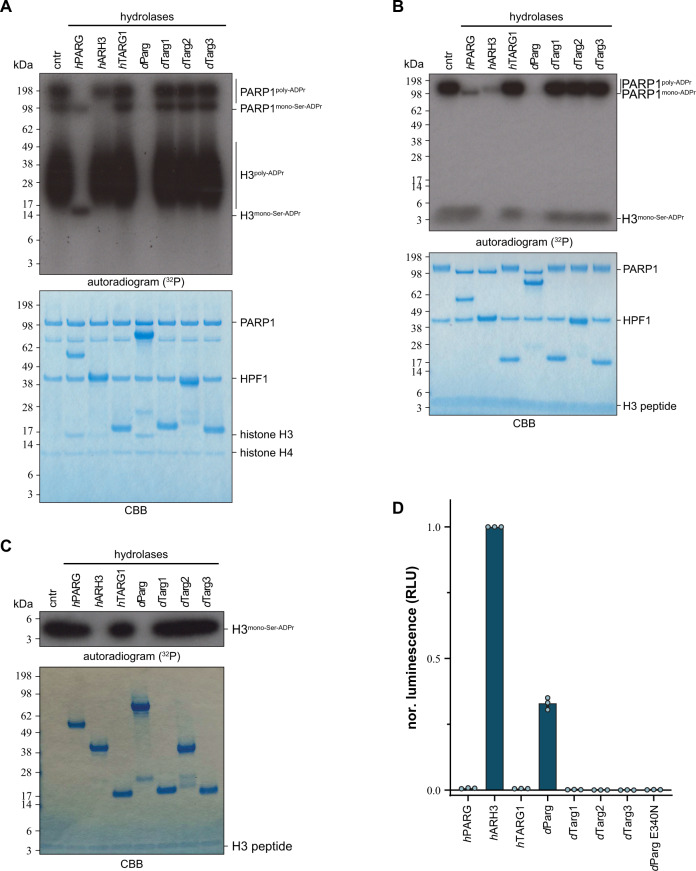
*d*Parg macrodomain catalyses mono-Ser-ADPr hydrolysis. **A** Removal of poly-Ser-ADPr on automodified *h*PARP1 in the presence of *h*HPF1 and poly-Ser-ADP-ribosylated histone H3/H4 tetramer by human and *Drosophila* (ADP-ribosyl)hydrolases. The reaction was performed as in Fig. 5A, except histone H3/H4 tetramer was used instead of histone H3 peptide. Lower panel shows CBB stained SDS-PAGE of the proteins. The experiment was repeated independently three times with similar results. **B** Removal of poly-ADPr on automodified *h*PARP1 (0.5 μM), in the presence of *h*HPF1 (0.5 μM) and mono-Ser-ADP-ribosylated H3 peptide (aa 1–21, 0.5 µg) by human and *Drosophila* (ADP-ribosyl)hydrolases. Reactions were performed as described in Fig. 5A. Lower panel shows the CBB stained SDS-PAGE of the proteins. The experiment was repeated independently three times with similar results. **C** Removal of mono-Ser-ADPr on mono-Ser-ADP-ribosylated histone H3 peptide (aa 1–21) by human and *Drosophila* (ADP-ribosyl)hydrolases. Reactions were performed as described in Fig. 1A and peptide purified as described in Fig. 1B. Lower panel shows CBB stained SDS-PAGE of the proteins. The experiment was repeated independently three times with similar results. **D** Measurements of hydrolase activity of indicated hydrolases against synthetic histone H2B peptide mono-Ser-ADPr on S7 using the AMP-Glo assay (Promega). Samples are background corrected and normalised to *h*ARH3. Data represent triplicate measurements of three independent experiments ± SEM.

As previously mentioned, *Drosophila* species lack ARH3 orthologues and hence cleavage of the (ADP-ribosyl)-seryl bond must be carried out by another enzyme. The persistence of the ADP-ribosylation signal in our experiments using PARGi in S2R+ cells suggests that *d*Parg is involved in DNA damage-dependent ADPr signal turnover (Figs. 2B, C, 3, and 4). Besides *d*Parg, *Drosophila* expresses three *h*TARG1 homologues (CG33054/*d*Targ1, CG33056/*d*Targ2 and CG34261/*d*Targ3^55^) whose potential contribution to Ser-ADPr removal cannot be ruled out. To assess the substrate specificity of these enzymes, we performed (ADP-ribosyl)hydrolase assays using different model substrates using the characterised human hydrolases *h*PARG, *h*ARH3 and *h*TARG1 as controls (Fig. 7 and Supplementary Fig. 5). Specifically, we utilised the previously established ability of *h*PARP1 WT to generate serine^15,18^- and glutamate^5,56^-linked poly-ADPr in presence and absence of *h*HPF1, respectively^30^. Likewise, we generated the mono-ADPr variants using the *h*PARP1 E988Q mutant, which is a specific mono-(ADP-ribosyl)transferase^57^. Both *h*PARG and *d*Parg readily removed ADP-ribose polymers, whereas the turnover by *h*ARH3 is less pronounced (Fig. 6A and Supplementary Fig. 5A), but were incapable of removing the terminal glutamate-ADPr linkage efficiently (Supplementary Fig. 5A, B). Furthermore, while *d*Parg and *h*ARH3 showed the ability to remove mono-Ser-ADPr from peptides and automodified *h*PARP, this was not seen for *h*PARG and the *Drosophila* TARG orthologues (Fig. 7).

When using serine-PARylated histone H3.1/H4 tetramer as substrate^13^, we observed both poly-Ser- and mono-Ser-ADP-ribosylated *h*PARP1, which allowed us to separately assess the activity of all tested (ADP-ribosyl)hydrolases against poly-Ser- and mono-Ser-ADPr (Fig. 7A). The assay clearly shows that *d*Parg efficiently reverses ADPr from both poly-Ser- and mono-Ser-ADP-ribosylated *h*PARP1 and histones. Conversely, *h*PARG only removed PAR from *h*PARP1 and histones, whereas *h*ARH3 efficiently removed mono-Ser-ADPr from *h*PARP1 but acted poorly on PAR and showed no activity against the modified histones. To compare the ability of *h*ARH3 and *d*Parg to remove mono-Ser-ADPr, we performed a hydrolases reaction utilising a synthetic histone H2B peptide, followed by conversion of the released ADP-ribose into AMP by human NudT5 and luminescence detection using the commercial AMP-Glo assay (Promega; Fig. 7D)^58–60^. Mono-Ser-ADPr hydrolysed by both *h*ARH3 and *d*Parg, albeit with *h*ARH3 more acting more efficiently. Together, our data show that complete Ser-ADPr reversal in *Drosophila* relies only on a single enzyme (*d*Parg), as opposed to the human system, which requires two enzymes (*h*PARG and *h*ARH3). Furthermore, the difference in mono-Ser-ADPr hydrolysis activity of *h*ARH3 and *d*Parg suggests that maintaining both catalytic functions – mono-Ser-ADPr and PAR hydrolysis – within a single protein (PARG) may come with an efficiency cost.

### Both PAR and Ser-ADPr hydrolysis are catalysed within the conserved active site of *d*Parg

**Fig. 8:**
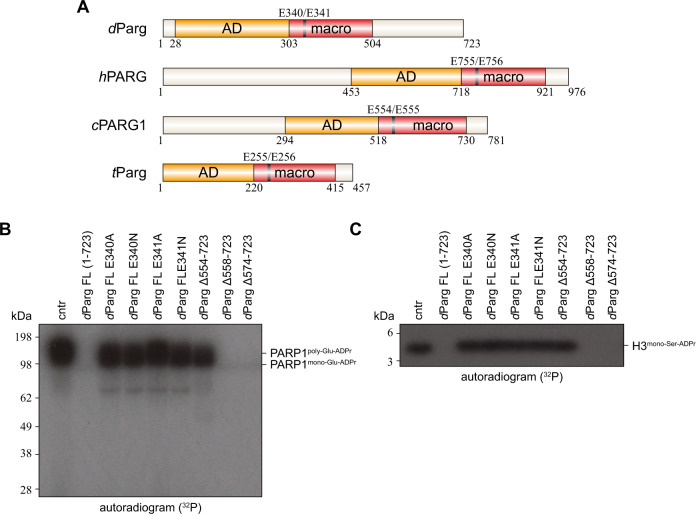
The mono-Ser-ADPr hydrolase activity of *d*Parg resides within its conserved active site. **A** Schematic representation of the PARG domain architecture. The catalytic domain is composed of two subdomains; an accessory domain (AD, yellow) and macrodomain (macro, red). Domain boundaries are given below and the catalytic EE motif (black line) above the diagram. Abbreviation *C. elegans* PARG1, *c*PARG1; *D. melanogaster* Parg, *d*Parg; *Homo sapiens* PARG, *h*PARG; *Tetrahymena thermophila* Parg, *t*Parg. **B** Activity of *d*Parg catalytic mutants and C-terminal truncations on poly-Glu-automodified *h*PARP1. Poly-Glu-automodified *h*PARP1 was obtained as described in Fig. 6C and subsequently supplemented with *d*Parg WT or with indicated mutants. The experiment was repeated independently three times with similar results. **C** Activity of *d*Parg catalytic mutants and C-terminal truncations on purified mono-Ser-ADP-ribosylated histone H3 peptide (aa 1–21). Mono-Ser-ADP-ribosylated histone H3 peptide (aa 1–21) was obtained as described in Methods and subsequently supplemented with *d*Parg WT or with indicated mutants. The experiment was repeated independently three times with similar results.

To investigate the unexpected ability of *d*Parg to remove both PAR chains as well as mono-Ser-ADPr, we compared its domain architecture to *h*PARG (Fig. 8A). While both enzymes share a conserved accessory and catalytic macrodomains motif, which in *h*PARG is responsible for PAR degradation, *d*Parg lacks the N-terminal region of *h*PARG and possesses an additional C-terminal domain (Fig. 8A). Therefore, we investigated whether this domain could be responsible for the hydrolysis of the serine-ribose linkage. To test this hypothesis, we generated three truncations of the C-terminal *d*Parg domain and assessed the ability of these variants to remove *h*PARP1 auto-PARylation (Fig. 8B) and histone H3 mono-Ser-ADPr (Fig. 8C). The three truncations showed that *d*Parg Δ554–723 lost activity against PAR and mono-Ser-ADPr, likely due to the contribution of the C-terminal domain towards the structural integrity of the enzyme, whereas *d*Parg Δ558–723 and *d*Parg Δ574–723 retained the ability to remove both PAR and mono-Ser-ADPr. These results show that the C-terminal extension of *d*Parg is not responsible for its specific Ser-ADPr activity and suggest that the conserved active site must be responsible for both PAR and Ser-ADPr removal activity.

Prior characterisation of PARGs identified a catalytic loop containing two absolutely conserved residues (Glu755 and Glu756 in humans) that are critical for the removal of PAR chains (Fig. 8A, B and Supplementary Fig. 6)^24^. We mutated the corresponding *d*Parg residues (Glu340 and Glu341) to both aspartate and alanine to assess whether these mutants would retain their ability to remove mono-Ser-ADPr. First, we assessed both WT and mutant *d*Parg against automodified *h*PARP1 (Fig. 8B). As expected, these mutations abolished *d*Parg activity against PAR. Likewise, we were unable to detect any *d*Parg activity against mono-Ser-ADP-ribosylated histone H3 (Fig. 8C). These data clearly show these mutations abolish *d*Parg activity, supporting the idea that the same active site is responsible for both PAR and mono-Ser-ADPr removal and leaving the question of how *d*Parg removes mono-Ser-ADPr.

### Protozoan *t*Parg removes mono-Ser-ADPr

**Fig. 9:**
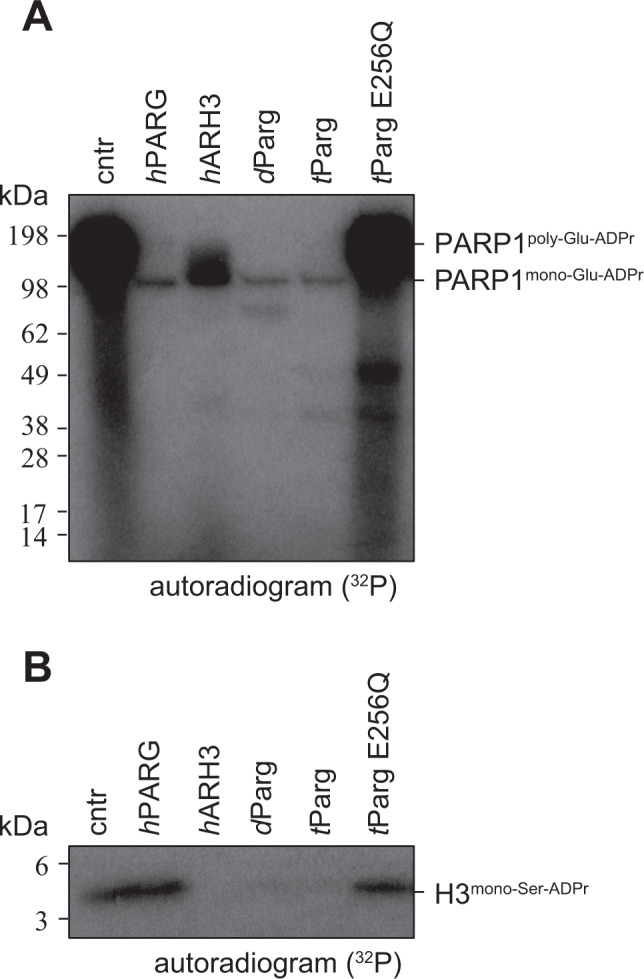
Protozoa *t*Parg removes mono-Ser-ADPr. **A** Activity of PARG homologue from *T. thermophila* against poly-Glu-automodified *h*PARP1. Poly-Glu-automodified *h*PARP1 was obtained as described in Fig. 6C. The experiment was repeated independently three times with similar results. **B** Activity of *t*Parg on purified mono-Ser-ADP-ribosylated histone H3 peptide (aa 1–21). Mono-Ser-ADP-ribosylated histone H3 peptide (aa 1–21) was obtained as described in the Methods. The experiment was repeated independently three times with similar results.

The discovery that the *d*Parg catalytic domain evolved Ser-ADPr removal activity prompted us to examine whether PARG homologs from other organisms could have such activity. We tested the activity of PARG homologs from the ciliate *Tetrahymena thermophila* (*t*Parg). In the absence of HPF1, the WT variants of all tested PARG are able to remove glutamate-linked PAR from automodified *h*PARP1, but leave a single band corresponding to mono-Glu-ADPr *h*PARP1 (Fig. 9A). The activity was abrogated in the catalytic *t*Parg E256Q mutant. Interestingly, *t*Parg behaves similarly to *d*Parg with regards to the removal of mono-Ser-ADPr from the modified H3 peptide, and this activity was lost in the catalytically dead *t*Parg E256Q mutant (Fig. 9B). These assays confirmed that the removal of mono-Ser-ADPr by PARG enzymes is not unique to *Drosophila*, but rather a mechanism shared by at least one other ARH3 lacking phylum (Fig. 1A).

### Crystal structure of *d*Parg

**Fig. 10:**
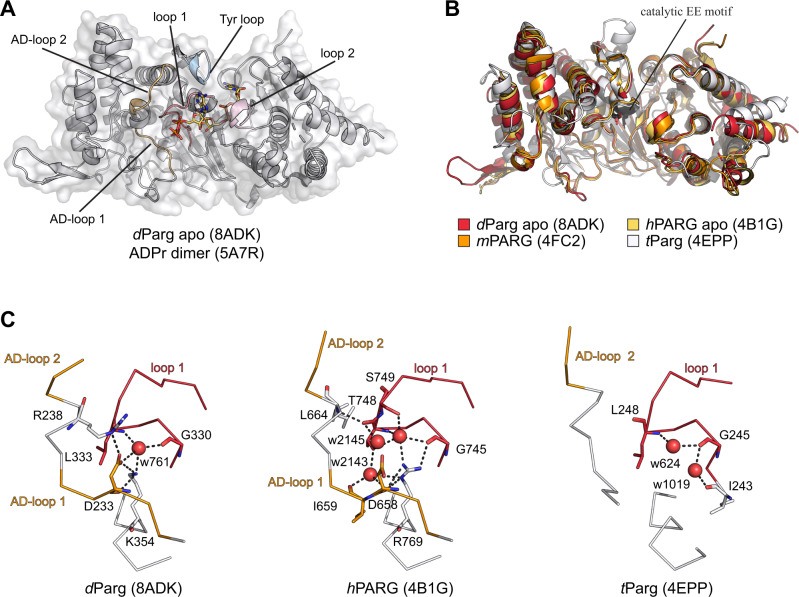
Structural basis of mono-Ser-ADPr hydrolysis by *d*Parg. **A** Ribbon-surface representation of apo *d*Parg. ADP-ribose dimer (yellow) of the aligned *h*PARG:ADP-ribose dimer complex structure is given to highlight the active site. Structural features important for catalysis are highlighted: accessory-domain loops 1 and 2, AD-loop 1 and 2; catalytic loop, loop 1; diphosphate binding loop, loop 2; tyrosine clasp, Tyr loop. **B** Ribbon representation of structural alignment of PARG domains from indicated species (*Drosophila*, red; human, yellow; mouse, orange; *T. thermophile*, white). The catalytic residues (Glu340/Glu341 in *d*Parg) are highlighted in black. **C** Ribbon-liquorice representation of loop 1-AD-loop 1 interaction of *Drosophila*, human, and *T. thermophile* PARGs. Polar interactions are highlighted as black dotted lines and water molecules involved in the interaction as red spheres.

To gain insights into the ability of *d*Parg to remove Ser-ADPr, we solved two structures of the catalytically active *d*Parg Δ574–723 truncation: the apo structure was solved to a resolution of 2.47 Å (PBD 8ADK, Fig. 10A and Supplementary Data 2) and the co-crystal structure with PARGi to a resolution of 2.51 Å (PDB 8ADJ, Supplementary Fig. 7 and Supplementary Data 2). Both structures are very similar with residues 26 to 525 and 533–547 clearly visible in the electron density and a RMSD of 0.139 Å over 369 aligned C^α^. The *d*Parg structure is composed of a central macrodomain fold that harbours the predicted substrate binding cleft as well as the catalytic residues^61,62^. The macrodomain is extended by a highly structured and conserved accessory domain so that the overall domain is composed of a twisted, mixed, ten-stranded β-sheet flanked by two predominantly α-helical sub-domains (Fig. 8A). The overall structure of *d*Parg is similar to other PARGs with RMSDs of 0.586 Å over 381 aligned C^α^ for human (PDB 4B1G), 0.651 Å over 366 aligned C^α^ for mouse (*m*PARG; PDB 4FC2), and 1.816 Å over 255 aligned C^α^ for *t*Parg (PDB 4EPP, Fig. 10B). Similarly, the *d*Parg:PARGi complex closely resembles *h*PARG complexes with similar inhibitors (RMSDs of 0.514 Å over 374 aligned C^α^ for the PDD00017262 [PBD 5LHB] and 0.491 Å over 369 aligned C^α^ for the PDD00017299 [PDB 6HML] complex). PARGi binding overlaps with the adenosine coordination region within the substrate binding cleft. The binding is tightly coordinated with staggered π-stacking interactions between Tyr380/Phe485 and the 2,4-quinazolinedione moiety (Supplementary Fig. 7) as well as polar interactions with the main chain of Ile311, Phe485 (Supplementary Fig. 7) and the side chains of Glu312, Gln339, and Phe485 (Supplementary Fig. 7). It is interesting to note that in the *h*PARG:ADPr complex (PDB 4NA0) Phe902, which is isostructural to *d*Parg Phe485, stacks with the adenine ring, which requires a side chain rotation of ~90° relative to the inhibitor stacking interaction. This shows that inhibitor binding not only competes for the binding space but also alters crucial substrate contacts.

The comparison of the *d*Parg with the mammalian *h*PARG and *m*PARG as well as protozoan *t*Parg structures shows a near identical active site architecture (Fig. 9B). The position of the catalytic loop (loop 1) is isostructural in the compared structures, while the diphosphate-binding loop (loop 2) is known to undergo conformational rearrangement upon substrate binding (Fig. 10B and Supplementary Figs. 7 and 8 and Supplementary Table 1)^63,64^. Loop 2 appears to crystalise in the open position and closely resembles the apo *m*PARG structure (Supplementary Fig. 8). In the *m*PARG:ADPr complex structure (PDB 4NA0) the loop moves slightly into the substrate binding cleft allowing the main chain nitrogen atoms of Gly866 and Ala867 to interact with the phosphate oxygen atoms of the ADP-ribose. Substrate binding is further accompanied by repositioning of Phe868 (Phe458 in *d*Parg) and His821 (His413 in *d*PARG), which are displaced from the binding cleft and contribute to the coordination of the distal ribose (Supplementary Fig. 7). These findings suggest that there are no major structural differences in coordination of the ADP-ribose moiety or placement of the catalytic residues and hint towards subtle differences between PARGs that can remove the terminal Ser-ADPr linkage and those that cannot.

Finally, we investigated structural features adjacent to loop 1: two loops located within the accessory domain were identified that both support loop 1 positioning as well as accessory-macrodomain interaction (termed AD-loop 1 and 2; Fig. 10A, B and Supplementary Fig. 6). The loop 1:AD-loop 1 interaction is stabilised by an extended water network in *h*PARG (Fig. 10C). However, these interactions are notably reduced in *d*Parg, while AD-loop 1 is absent in *t*Parg (Fig. 10C). The main difference in the coordination network between loop1 and AD-loop 1 is the presence of a threonine residue (Thr748 in *h*PARG, whereas both *d*Parg and *t*Parg contain a leucine residue (Leu333 and Leu248, respectively) in the isostructural position. Our phylogenetic analysis showed that threonine is conserved within the *Mammalia* and the leucine can be found in *Diptera*, *Nematoda* and *Protozoa* (Supplementary Fig. 6), thus suggesting that it is indeed one factor in the substrate specificity. However, the distance from the catalytic EE motif as well as its orientation away from the substrate (Fig. 10A) suggests no direct involvement in the catalytic mechanism.

## Discussion

Serine-linked ADP-ribosylation is a crucial signalling mechanism in the DDR of humans and other mammalian species. Here we provided evidence that this signalling variant is spread throughout the *Animalia* and may be a defining feature of the DDR regulation of this kingdom. Using cutting-edge mass spectrometry, we provide a first draft of the *Drosophila* ADP-ribosylome identifying > 500 high confident ADPr sites. Previously, ADPr has been reported to modify aspartic acid, glutamic acid, lysine, and arginine residues^52,65,66^. The relatively recent discovery of serine residues as acceptor sites^13^, has led to the identification of serine as the most abundantly modified amino acid residue under DNA damage in cell culture^12,14^. By combining the Af1521 enrichment strategy, which is able to identify ADPr on all possible amino acid residues^50,67,68^, with ETD fragmentation for proper localisation of the modification site^12^, we identified serine as the most abundantly modified residue in *Drosophila* under these experimental conditions. Still, experimental conditions as well as the depth of sequencing could cause the absence of other known amino acid acceptor residues. Our analysis of the Ser-ADPr cycle in *D. melanogaster* further revealed a striking conservation with the human signalling pathway. On the molecular level not only the mammalian ADPr consensus motif ‘KS’ is conserved, but we observe a broad overlap with previously identified ADPr targets in humans. This is particularly true for the main ADP-ribose acceptors such as PARP1 and histones. In both species, pathways relevant for genome stability, chromatin structure regulation, and transcription are major targets for this modification. Thus, our data suggests that *Drosophila* can serve as a model organism to provide insights into the physiological function of Ser-ADPr signalling. This includes the possibility of understanding the links between this modification and associated diseases including neurodegeneration and cancer^30,41,69–71^. In this respect, it was previously shown that *d*Parg deficiency could be complemented using human *ARH3* gene^71^. Also, as the *h*PARP1 automodification region that has been shown to be important for *h*PARP1 trapping at DNA breaks and the PARP inhibitor response in humans is functionally conserved in *Drosophila* species (Fig. 4G and Supplementary Fig. 4), this model could be useful for understanding the physiological effects of clinically relevant PARP inhibitors^30^.

Our phylogenetic analysis highlights that amongst HPF1 carrying species, ARH3 is absent in *Protozoa*, *Nematoda*, *Lepidoptera* and *Diptera*. In contrast, ARH3 can be identified in most *Animalia*, including basal ones from the *Placozoa*, *Porifera*, or *Cnidaria* phyla. This pattern of presence and absence of ARH3 strongly suggests an evolutionary history that (i) contains a gain of ARH3 in the early evolution of *Animalia* and (ii) at least two independent loss events: first in the split between *Nematoda* and *Arthropoda*, and second during diversification within the *Endopterygota* superorder. Interestingly, PARP2, which in humans can also generate Ser-ADPr, is also absent in *Drosphila*. Hence, it appears that *Drosophila*, despite the conservation of physiological function, utilises only a minimal Ser-ADPr system for the regulation of the DDR consisting of *d*HPf1, *d*Parp as the only DNA repair PARP (albeit with *h*PARP1 domain architecture), as well as *d*Parg, which combines both poly- and mono-Ser-(ADP-ribosyl)hydrolase activity. Given the functional similarities, this may be advantageous for some studies as it allows easier manipulation of signal establishment and removal. Furthermore, our study revealed that tools developed for the study and clinical application of human Ser-ADPr, such as ADPr detection reagents and antibodies as well as inhibitors, can be applied to the study of ADPr signalling in *Drosophila*. Our structural data revealed that the active site of *d*Parg is conserved with respect to mammalian and protozoan PARGs, hence indicating that the difference in activity is not a result of an altered catalytic mechanism. Together our data suggests that the ability to cleave the Ser-ADPr bond relies on subtle structural differences surrounding the active site that may (dis)allow access of certain substrates. This idea is further supported by the differences in substrate geometry. Structural data of an ADP-ribose dimer in complex with *h*PARG (PDB 5A7R) indicate that the *n*−1 unit extends linearly out of the active site (Fig. 10A). In contrast, the serine modified peptide co-crystallised with *h*ARH3 (PDB 7AKS) lies perpendicular to the ADP-ribose binding pocket. However, further studies are needed to discern the mode of interaction of different PARGs with their various substrates. Based on our phylogenetic and biochemical findings, it is interesting to speculate that the ability of PARGs to cleave the terminal protein-ribose linkage may not be limited to fruit flies. This is supported by (i) the identification of several evolutionary branches that carry HPF1, but lack ARH3 (Fig. 1), (ii) our experimental confirmation that *t*Parg can also remove Ser-ADPr (Fig. 9) as well as (iii) recent observations in plants showing that *Arabidopsis thaliana* Parg1 can remove mono-ADPr from SZF1^72^. Notably, *PARG* gene duplications have been described in both plants and *C. elegans*^73,74^, which is indicative of a diversification of known ADPr signalling systems which may hold new surprises for future discoveries.

## Methods

### Cell culture

The *Drosophila* S2R+ cell line was purchased in DGRC (https://dgrc.bio.indiana.edu/Home) and were cultured in *Drosophila* Schneider’s media (21720-024, Gibco) supplemented with 10% heat-inactivated fetal bovine serum (10500-056, Gibco) and 1% penicillin-streptomycin (100 U/ml, 15140-122, Gibco) at 25 °C and passaged every 3–4 days. The human U2OS osteosarcoma cell line was purchased in ATCC (HTB-96) and were grown in DMEM (10566016, Gibco) supplemented with 10% FBS (F9665, Sigma) and penicillin-streptomycin (100 U/mL, GIBCO) at 37 °C with 5% CO2 and passaged every 3–4 days. For all DNA damage induction experiments, cells were seeded at a density of 5 × 10^6^ cells for S2R+ cells or 2 × 10^6^ cells for U2OS cells in a 6 cm dish. The next day cells were once carefully washed with PBS and damaged with 2 mM H_2_O_2_ (H1009, Sigma) or 5 mM MMS (129925, Sigma) in PBS plus calcium and magnesium (DPBS, Gibco, 14040-133) for the indicated times. For treatment of PARG inhibitor, 5 × 10^6^ cells were seeded in a 6 cm dish. The next day cells were treated with 2 μM PARG inhibitor (PDD00017273, Sigma) for 16 h, whereas control cells were treated with DMSO. This was followed by H_2_O_2_ treatment as described above.

### Immunoblot

Cells were lysed in 50 mM TrisHCl (pH 8.0), 100 mM NaCl, and 1% (v/v) Triton X-100, 5 mM MgCl_2_, 1 mM DTT, supplemented with 1× Protease inhibitor (Roche), 1 μM PARG inhibitor (PDD00017273, Sigma), and 1 μM PARP inhibitor (Olaparib, LKT LABS). The lysates were incubated with 0.1% benzonase (Sigma) for 30 min at 4 °C. The soluble fraction was mixed with NuPAGE LDS sample buffer (Invitrogen) with 50 mM DTT and proteins were denatured at 95 °C for 5 min. The whole cell extracts from S2R+ cells were electrophoretically separated on NuPAGE Novex 4–12% Bis-Tris gels (Invitrogen) and transferred to nitrocellulose membranes (Bio-Rad) for 30 min using Trans-Blot Turbo Transfer System (Bio-Rad). The blotted membranes were blocked with PBS buffer containing 0.1% (v/v) Tween 20 and 5% (w/v) skimmed milk powder for 1 h at room temperature and then incubated with rabbit anti-poly ADPr antibody (4336-BPC-100, Trevigen, 1:1,000, RRID: AB_2721257), rabbit anti-poly ADPr anti reagent (MABE1031, Millipore, 1:500, RRID: AB_2665467), rabbit anti-pan ADPr anti reagent (MABE1016, Millipore, 1:1,000, RRID: AB_2665466), rabbit anti-mono ADPr anti reagent (MABE1076, Millipore, 1:500, RRID: AB_2665469), rabbit anti-mono ADPr antibody (AbD33204, BioRad, 1:1,000), rabbit anti-phosphor Histone H2AvD (Ser137) antibody (600-401-914, Rockland, 1:3,000, RRID: AB_828383), mouse anti-phosphor Histone H2A.X (Ser139) antibody (clone JBW301, 05-636, Millipore, 1:500, RRID: AB_309864) or mouse anti-actin monoclonal antibodies (JLA20, concentration, Developmental Studies Hybridoma Bank, 1:10,000, RRID: AB_528068) at 4 °C overnight. After washing with PBS containing 0.1% (v/v) Tween 20, the blots were incubated with a horseradish peroxidase-labelled anti-rabbit IgG (P0399, Dako, 1:4,000, RRID: AB_2617141) or anti-mouse IgG (P0447, Dako, 1:4,000, RRID: AB_2617137) for 1 h. Detection was performed using Pierce ECL Western blotting substrate (Thermo Scientific) and analysed by luminography using Hyperfilm ECL (Amersham). Experiments were conducted for a minimum of three independent repeats.

### Mass spectrometry

#### Cell lysis and purification of ADP-ribosylated peptides

ADP-ribosylated peptides were lysed and enriched as described previously ref. ^12, 51, 75^. In brief, cell pellets were lysed in 10 pellet volumes of Lysis Buffer (6 M guanidine hydrochloride, 50 mM TrisHCl [pH 8.5]), and complete lysis was achieved by alternating vigorous shaking with vigorous vortexing. Upon reduction and alkylation using TCEP and CAA, proteins were digested using Lysyl Endopeptidase (Lys-C, 1:100 w/w; Wako Chemicals) for 3 h and diluted with three volumes of 50 mM ammonium bicarbonate. Samples were further digested overnight using modified sequencing grade Trypsin (1:100 w/w; Sigma Aldrich). Digested samples were purified using reversed-phase C18 cartridges according to the manufacturer’s instructions. Elution of peptides was performed with 30% ACN in 0.1% TFA, peptides were frozen overnight at −80 °C, and afterwards lyophilised for 96 h.

Lyophilised peptides were dissolved in AP buffer (50 mM TrisHCl [pH 8.0], 1 mM MgCl_2_, 250 μM DTT, and 50 mM NaCl), and ~2 mg of peptide was used for each replicate experiment. Samples were incubated with Af1521 and left head-over-tail rotating at 4 °C for 4 h. The beads were washed twice in freshly prepared ice-cold AP buffer, twice in ice-cold PBS with DTT, and twice in ice-cold MQ water, with a tube change every time the buffer was changed. ADPr-modified peptides were eluted off the beads by addition of ice-cold 0.15% TFA. Eluted peptides were passed through 0.45 μm spin filters, and afterward through pre-washed 100 kDa cut-off spin filters (Vivacon 500, Satorius), after which they were high pH fractionated into three fractions and an additional F0^12,50,51,53^.

#### Mass spectrometric analysis and data analysis

All MS experiments were analysed on an EASY-nLC 1200 HPLC system (Thermo) connected to a Fusion Lumos Orbitrap mass spectrometer (Thermo). Each sample was separated on a 15 cm analytical column, with an internal diameter of 75 μm, packed in-house with 1.9 μm C18 beads (ReproSil-Pur-AQ, Dr. Maisch), and heated to 40 °C using a column oven. Peptide separation was performed using a 60 min gradient at a flow rate of 250 nL/min, utilising buffer A consisting of 0.1% FA, and buffer B consisting of 80% ACN in 0.1% FA. The mass spectrometer was operated in data-dependent acquisition mode, with full scans performed at a resolution of 120,000 and a maximum injection time of 250 ms. Precursor fragmentation was accomplished using electron transfer disassociation with supplemental higher-collisional disassociation (EThcD), with supplemental activation energy of 20. Precursors with charge state 3–5 were included and prioritised from charge 3 (highest) to charge 5 (lowest), using the decision tree algorithm. Selected precursors were excluded from repeated sequencing by setting a dynamic exclusion of 45 s. MS/MS spectra were measured in the Orbitrap, with a maximum precursor injection time of 500 ms, and a scan resolution of 60,000. All MS raw data were analysed using the MaxQuant software suite version 1.5.3.30^76^, and searched against the *Drosophila* proteome in FASTA file format, as downloaded from UniProt on the 11^th^ of November 2020. Default MaxQuant settings were used except the following: cysteine carbamidomethylation, and ADP-ribosylation on cysteine, aspartic acid, glutamic acid, histidine, lysine, arginine, serine, threonine, and tyrosine residues were included as variable modifications. The Andromeda delta score was set to minimum 20 for modified peptides.

Statistical handling of the data was primarily performed using the freely available Perseus software^77^, and includes principal component analysis and volcano plot analysis. Protein Gene Ontology annotations were performed using DAVID Bioinformatics Resources^78^. Sequence context analysis was performed using iceLogo software^79^.

### RNA interference

RNA interference analysis was carried out as previously described in ref. ^80, 81^. The nucleotides 34–367 of d*Parg* cDNA were chosen as the targets of ds*PARG-1* using SnapDragon (https://www.flyrnai.org/cgi-bin/RNAi_find_primers.pl). The targets of ds*PARG-2* (769-1275) and ds*LacZ* were produced as previously described in ref. ^32^. Oligonucleotides to generate templates for dsRNAs by PCR are given in Supplementary Data 3. dsRNAs were prepared using MEGAscript T7 kit (Thermo Fisher Scientific, AM1334) according to the manufacturer’s instructions. The RNA was denatured at 65 °C for 30 min and then annealed by slowly cooling down to 4 °C. 10 μg of dsRNA was added per 1 × 10^6^ cells. Cells were harvested for 5 days after dsRNA treatment, followed by induction of the DNA damage using H_2_O_2_ as described above or reverse transcriptase-Quantitative polymerase chain reaction (RT-qPCR) analysis as described below.

### RT-qPCR

The total RNAs from S2R+ cells were purified with RNeasy Plus Mini kit (QIAGEN) and then 0.5 μg total RNA was used for cDNA synthesis with QuantiTect Reverse Transcription Kit according to the manufacturer’s instructions. The cDNAs were detected by quantitative real-time PCR using the Rotor-Gene SYBR Green PCR Kit and the Rotor-Gene Q (QIAGEN). Primer pairs for RT-qPCR are given in Supplementary Data 3. The relative gene expression analysis of d*Parg* gene was performed using the ddCt method.

### Quantification and statistical analysis

Prism 9.1 (GraphPad) was used for statistical analysis, where ****p < 0.0001. Details of statistical analyses are described in the Fig. 4 legend.

### Cloning, expression, and purification

Expression vectors for *h*ARH3, *h*TARG1, *h*HPF1, *h*PARP1 and *h*PARG were described earlier^5,15,54,63^. The coding sequence of *d*Parp, *d*Parg, *d*Targ1-3 and *d*Hpf1 were amplified from cDNA prepared from S2R+ cells using oligonucleotides listed in Supplementary Data 3 and cloned into pET28a expression plasmids with an N-terminal His-tag. All indicated mutations were introduced via PCR based site-directed mutagenesis (Supplementary Data 3). Expression was carried out in *E. coli* Rosetta (DE3) cells (Novagen), and Terrific Broth media supplemented with 30 μg/ml kanamycin and 30 μg/ml chloramphenicol. Cells were grown at 37 °C and growth stopped when cultured reached OD_600_ ~ 0.6. Cultures were then induced with 1 mM IPTG and incubated at 18 °C overnight. Cells were centrifuged for 10 min at 3000 x g, and the pellets resuspended in buffer A (50 mM TrisHCl (pH 8.0), 150 mM NaCl, 1 mM TCEP, 10 mM imidazole) supplemented with 1× cOmplete EDTA free protease inhibitor cocktail (Roche) and 250 U of benzonase nuclease (Sigma) per 1 L of cell culture. All the following purification steps were performed at 4 °C. Lysis was performed using a homogeniser, and cell debris separated by centrifugation at 35,000 x g for 60 min. The supernatant was then incubated with Ni-NTA resin (Qiagen) pre-equilibrated with buffer A, for 30 min. The suspension was transferred into an empty gravity flow column (BioRad), and the resin washed with 10 column volume of buffer A prior to elution with buffer A supplemented with 300 mM imidazole. Eluted proteins were dialysed against 25 mM TrisHCl (pH 8), 500 mM NaCl and 1 mM DTT at 4 °C, overnight. The proteins were then concentrated and subjected to size-exclusion chromatography using a HiLoad 16/60 Superdex 75 column equilibrated with 10 mM TrisHCl (pH 8), 100 mM NaCl, 0.2 mM TCEP for *d*Parg and *d*Targ1-3, or 10 mM TrisHCl (pH 8), 100 mM NaCl, 0.1 mM TCEP for *d*Targ1-3 and *d*Hpf1, respectively. Eluted *d*Parg and *d*Targ1-3 were concentrated to 8 mg/ml and *d*Hpf1 to 9 mg/ml. Protein quality was assessed for each step by SDS-PAGE. All other proteins were expressed and purified as described previously: *h*PARG^62^, *h*HPF1^15^, *h*PARP1 wild type and the E988Q mutant^82^, *h*ARH1, *h*ARH2, *h*ARH3^83^, and histone H3/H4^84^. histone H3 peptide (aa 1–21) was purchased from Sigma (SaintLouis, MO, US).

### In vitro (ADP-ribosyl)hydrolase assays

#### Demodification of enzymatically generated ADP-ribosyl modification

ADPr was performed as previously described^25^. Briefly, recombinant proteins or peptides were ADP-ribosylated by *h*PARP1 to produce Glu-ADPr or by *h*PARP1:*h*HPF1 to produce Ser-ADPr. The reactions were performed in 50 mM TrisHCl (pH 8), 100 mM NaCl, 2 mM MgCl_2_, activated DNA and 50 µM NAD^+^ spiked with ^32^P-NAD^+^. The *h*PARP1 reaction was performed at room temperature for 30 min and stopped by the addition of 1 µM olaparib. ADP-ribosylated proteins were used as the substrate for the successive (ADP-ribosyl)hydrolase assays. The substrate was incubated at room temperature for 30 min with the indicated (ADP-ribosyl)hydrolases and analysed by SDS-PAGE and autoradiography. *h*PARP1 and *h*HPF1 concentrations per reaction were 0.5 μM, (ADP-ribosyl)hydrolase was 1 μM, histone tetramere H3/H4 2 μM and histone peptides 0.5 µg.

#### Detection of (ADP-ribosyl)hydrolase activity by AMP-Glo assay

The assay was performed as previously described^58–60^. Briefly, the concentration of the synthetic mono-Ser-ADPr H2B peptide^59^ was estimated using absorbance at λ_260nm_ with a molar extinction coefficient of 13,400 M^−1^ cm^−1^ for the ADP-ribosyl modification. 8 μM peptide was demodified by incubation with 1 μM indicated hydrolase for 30 min at 30 °C in assay buffer (50 mM TrisHCl [pH 8], 200 mM NaCl, 10 mM MgCl_2_, 1 mM dithiothreitol and 0.2 μM human NudT5^85^). Reactions were stopped and analysed by performing the AMP-Glo™ assay (Promega) according to the manufacturer’s protocol. Luminescence was recorded on a SpectraMax M5 plate reader (Molecular Devices) and data analysed with GraphPad Prism 9.1. For background subtraction reaction were carried out in the absence of hydrolase.

### Purification of Ser-ADP-ribosylated histone peptide

Histone H3 peptide was Ser-ADP-ribosylated as above, except that higher concentrations of substrate were used. Ser-ADP-ribosylated peptides were further purified by filtering the reaction using a concentration column with a 10 kDa cut-off (Millipore). Excess NAD^+^ was removed using a G25 spin column (GE HealthCare, UK).

### Inference of phylogenetic relationships and sequence similarities

Alignments of HPF1 sequences from metazoan and protozoan species (Supplementary Data 1) were generated using JalView v. 2.11^86^ and the HPF domain extracted from their sequential context based on Mafft L-INS-i alignment^87^ using crystallographic data to determine domain boundaries. Extracted sequences were re-aligned using Mafft L-INS-i algorithm. The evolutionary histories of the HPF domain was inferred by using the maximum-likelihood method and Le_Gascuel_2008 model^88^ with an automatically obtained initial tree for the heuristic search by applying the maximum-parsimony method. The analysis was carried out using a site coverage of 95% with partial-deletion option. Confidence levels were estimated using 1000 cycles of the bootstrap method. Evolutionary analyses were conducted in MEGA11^89^.

Alignments of PARP and PARG sequences (Supplementary Tables 1 and 3) were generated using JalView v. 2.11 using the implemented Mafft L-INS-i algorithm.

### Crystallisation, data collection, structure solution, refinement, and analysis

Crystallisation trials were performed at 4 °C with commercial screens using the vapor diffusion method with the aid of a Mosquito Crystal robot (TTP Labtech) using sitting drops of 150 nl protein solution in MRC two-well crystallisation microplates (Swissci) equilibrated with 150 nl reservoir. Crystals of *d*Parg were grown in 19% (w/v) PEG3350, 210 mM sodium sulphate, 0.1 M Bis-Tris propane (pH 7.2). Crystals of *d*Parg in complex with inhibitor PDD00017273 grew in the same condition, except that PDD00017273 was added to the protein solution to a concentration of 0.5 mM prior to crystallisation. All crystals were cryoprotected in 15% (v/v) glycerol in the mother liquor before being vitrified by submersion in liquid nitrogen. Data collection was performed at beamlines I04 and I24 of the Diamond Light Source (Rutherford Appleton Laboratory, Harwell, UK).

X-ray data were processed using Xia2^90^. PHASER^91^ was used for molecular replacement trials with *h*PARG (PDB: 6HMK) as molecular replacement model. Density modification was performed with PARROT^92^ and initial models were built using the automated model building program BUCCANEER^93^. Model building for all structures were carried out with COOT^94^ and real space refinement with REFMAC5^95^, coupled with automatically generated local non-crystallographic symmetry restraints and TLS refinement. Statistics for *d*Parg and *d*Parg:PDD00017273 complex are shown in Supplementary Table 2.

### Live-cell microscopy

*Drosophila* S2R+ cells were plated on an 8-well ibiTreat chamber slide (ibidi) and transfected 48 h prior to imaging using FugeneHD according to the manufacturer’s instructions. For cell sensitisation prior to laser irradiation at 405 nm, growth medium was aspirated from the chamber slide and replaced with fresh medium containing 0.3 μg/mL Hoechst 33342. Immediately prior to imaging, the Hoechst containing media was replaced with fresh growth media. Live-cell microscopy was carried out on an Olympus IX-83 inverted microscope equipped with a Yokogawa SoRa super-resolution spinning-disk head, a UPlanAop 60x/1.5 N.A. oil-immersion objective lens for microirradiation experiments, a UPlanXApo 100×/1.35 N.A. for protein localisation experiments and a Prime BSI sCMOS camera. The fluorescence of Hoechst and EGFP were excited with 405 nm and 488 nm solid state laser respectively and fluorescence detection was achieved with bandpass filters adapted to the fluorophore emission spectra. Laser microirradiation at 405 nm was performed along a 7 µm line through the nucleus for 250 ms using a single-point scanning head (Olympus cellFRAP) coupled to the epifluorescence backboard of the microscope. To ensure reproducibility laser power at 405 nm was measured at the beginning of each experiment and set to 110 µW at the sample level. For time-course experiments, images were collected every 2 s. For the live-cell imaging experiments, cells were maintained at 25 °C with a heating chamber. Protein accumulation at sites of damage (*A*_*d*_) was then calculated as:

$${A}_{d}=\frac{{I}_{d}-{I}_{{bg}}}{{I}_{n}-{I}_{{bg}}}$$

The intensity within the microirradiated area was then normalised to the intensity prior to damage induction.

For protein localisation images, *Drosophila* S2R+ cells transfected with EGFP-tagged proteins were incubated in media containing 1 μg/mL Hoechst 33342 for 30 min. Hoechst containing media was replaced with fresh growth media prior to imaging.

### Reporting summary

Further information on research design is available in the Nature Portfolio Reporting Summary linked to this article.

## Supplementary information

Supplementary Information

Description of Additional Supplementary Files

Supplementary Data 1

Supplementary Data 2

Supplementary Data 3

Reporting Summary

## Data Availability

The atomic coordinates included in the study have been deposited in the Protein Data Bank (PDB) with the following accession codes: apo *d*Parg, 8ADK [10.2210/pdb8adk/pdb]; *d*Parg:PARGi complex, 8ADJ [10.2210/pdb8adj/pdb]. The mass spectrometry proteomics data have been deposited to the ProteomeXchange Consortium via the PRIDE partner repository^96^ with the dataset identifier PXD036512. Full images of the blots and gel as well as data used to generate graphs can be found in the Source data file. Source data are provided with this paper.

## Source data

Source Data

## Materials availability

All constructs generated in this study are available upon request and will be fulfilled by the Lead Contact with a completed Materials Transfer Agreement.
