# Supplementary material for: Serine ADP-ribosylation in Drosophila provides insights into the evolution of reversible ADP-ribosylation signalling
Source: Nat Commun. 2023 Jun 2;14:3200. doi: 10.1038/s41467-023-38793-y (PMC10238386; doi:10.1038/s41467-023-38793-y)
Supplement: Supplementary file 1 — Supplementary Information [file 41467_2023_38793_MOESM1_ESM.pdf]

Supplemental Information

Serine ADP-ribosylation in *Drosophila* provides insights into the evolution of reversible ADP-ribosylation signaling

Pietro Fontana[1,2,3,*], Sara C. Buch-Larsen[4,*], Osamu Suyari[1,*], Rebecca Smith[1], Marcin J. Suskiewicz[1,5], Kira Schützenhofer[1], Antonio Ariza[1,6], Johannes Gregor Matthias Rack[1,7], Michael L. Nielsen[4], Ivan Ahel[1]

[1] Sir William Dunn School of Pathology, University of Oxford, South Parks Road, Oxford, OX1 3RE, UK.

[2] Department of Biological Chemistry and Molecular Pharmacology, Harvard Medical School, Boston, MA, USA.

[3] Program in Cellular and Molecular Medicine, Boston Children's Hospital, Boston, MA, USA.

[4] Proteomics program, Novo Nordisk Foundation Center for Protein Research, Faculty of Health and Medical Sciences, University of Copenhagen, Blegdamsvej 3B, 2200 Copenhagen, Denmark.

[5] Current address: Centre de Biophysique Moléculaire, UPR4301 CNRS, rue Charles Sadron, CEDEX 2, F-45071 Orléans, France.

[6] Current address: School of Biosciences, University of Sheffield, Western Bank, Sheffield, S10 2TN, UK.

[7] Current address: MRC Centre for Medical Mycology, School of Biosciences, University of Exeter, Geoffrey Pope Building, Exeter, EX4 4QD, UK.

*These authors contributed equally.

Correspondence to:
Ivan Ahel (ivan.ahel@path.ox.ac.uk)
Michael L. Nielsen (michael.lund.nielsen@cpr.ku.dk)
Johannes G. M. Rack (j.rack@exeter.ac.uk)
Antonio Ariza (a.ariza@sheffield.ac.uk)

Supplemental Table 1. PARP sequences utilized to analyse automodification domain.

species				
binominal name	common name[a]	accession[b]	ARH3 homologue present	HPF1 with PBZ
Insecta				
Acromyrmex heyeri	Formiga-de-monte-vermelha	KAG5332370	yes	yes
Aphis gossypii	Cotton aphid	XP_027836345	no	yes
Apis mellifera	Honey bee	XP_624477	yes	yes
Athalia rosae	Coleseed sawfly	XP_012253348	yes	yes
Bemisia tabaci	Silverleaf whitefly	CAH0393176	yes	yes
Blattella germanica	German cockroach	PSN54639	yes	yes
Bombyx mori	Domestic silk moth	XP_004926518	no	yes
Cimex lectularius	Bed bug	XP_014252361	yes	yes
Cloeon dipterum	Pond Olive	CAB3383108	no	no[c]
Cryptotermes secundus	*n.a.*	XP_023719718	yes	yes
Danaus plexippus plexippus	Monarch Butterfly	OWR47253	no	yes
Diabrotica virgifera virgifera	Western corn rootworm	XP_028137801	yes	yes
Drosophila melanogaster	Fruit fly	NP_001104452	no	yes
Formica exsecta	Narrow-headed ant	XP_029670266	yes	yes
Frankliniella occidentalis	Western flower thrips	XP_026271855	yes	yes
Galleria mellonella	Greater wax moth	XP_026751130	no	yes
Harmonia axyridis	Halloween beetle	XP_045461901	yes	yes
Homalodisca vitripennis	Glassy-winged sharpshooter	XP_046681652	yes	yes
Hyposmocoma kahamanoa	*n.a.*	XP_026328627	no	yes
Ignelater luminosus	Cucubano	KAF2885554	yes	yes
Ischnura elegans	Blue-tailed damselfly	XP_046383121	yes	yes
Lasius niger	Black garden ant	KMQ98224	yes	yes
Monomorium pharaonis	Pharaoh ant	XP_012538492	yes	yes
Musca domestica	Housefly	XP_005175605	no	yes
Myzus persicae	Green peach aphid	XP_022178738	no	yes
Ooceraea biroi	Clonal raider ant	XP_011334668	yes	yes
Photinus pyralis	Common eastern firefly	XP_031330927	yes	yes
Schistocerca americana	American grasshopper	XP_046979867	yes	yes
Sipha flava	Yellow sugarcane aphid	XP_025410796	no	yes
Sitophilus oryzae	Rice weevil	XP_030752809	yes	yes
Thrips palmi	Melon thrips	XP_034248574	no	yes
Timema tahoe	*n.a.*	CAD7458304	yes	yes
Tribolium castaneum	Red flour beetle	EFA00829	yes	yes
Tribolium madens	Black flour beetle	XP_044269707	yes	yes
Trichoplusia ni	Cabbage looper	XP_026729426	no	yes
Zerene cesonia	Dogface butterfly	XP_038210310	no	yes
Mammalia				
Balaenoptera musculus	Blue whale	XP_036723058	yes	no
Homo sapiens	Human	NP_001609	yes	no
Orycteropus afer afer	Aardvark	XP_007935170	yes	no

[a]not applicable (*n.a.*) designates species with no common name
[b]GenBank accession number
[c]HPF domain is predicted to be part of a multidomain protein (GenBank CAB3360198) with unique architecture. Our analysis of the genomic region revealed the presence of a potential PBZ coding region upstream of the HPF domain.

Supplemental Table 2. Data collection and refinement statistics (molecular replacement)

	*d*Parg[a] (PDB 8ADK)	*d*Parg:PDD00017273[a] (PDB 8ADJ)
**Data collection**		
Space group	$P\,2_1$	$P\,2_1$
Cell dimensions		
a, *b*, *c* (Å)	93.43, 115.62, 122.91	93.39, 115.90,2 123.27
α, β, γ (°)	90.00, 90.00, 112.21	90.00, 90.00, 112.21
Resolution (Å) [b]	58.95 – 2.48 (2.53 – 2.48)	59.02 – 2.51 (2.58 – 2.51)
R_{sym} or R_{merge}[b]	0.087 (1.116)	0.133 (2.020)
$I / \sigma I$[b]	9.6 (1.1)	9.6 (1.1)
Completeness (%)[b]	99.7 (99.3)	100.0 (100.0)
Redundancy[b]	3.3 (3.4)	6.7 (6.7)
**Refinement**		
Resolution (Å)	2.47	2.51
No. reflections	85715	83225
R_{work} / R_{free}	0.177 / 0.228	0.183 / 0.223
No. atoms		
Protein	12576	12521
Ligand/ion	63/3	165/3
Water	277	201
B-factors		
Protein	70.57	72.70
Ligand/ion	85.17/75.39	79.27/81.75
Water	51.91	55.00
R.m.s. deviations		
Bond lengths (Å)	0.0123	0.0092
Bond angles (°)	1.895	1.681

[a]Data were collected from a single crystal

[b]Values in parentheses are for highest-resolution shell.

Supplemental Table 3. PARG sequences utilized for multiple sequence alignment.

species		
**binominal name**	**common name**[a]	**accession number**[b]
Caenorhabditis elegans	*n.a.*	NP_001023135[c]
Caenorhabditis elegans	*n.a.*	NP_501496[d]
Drosophila bunnanda	*n.a.*	KAH8234568
Drosophila melanogaster	Fruit fly	NP_001245511
Felis catus	Domestic cat	XP_011285559
Homo sapiens	Human	NP_003622
Loa loa	Eye worm	XP_020303580
Monosiga brevicollis MX1	*n.a.*	XP_001748857
Mus musculus	Mouse	NP_036090
Musca domestica	House fly	XP_005174952
Tetrahymena thermophila	*n.a.*	XP_001013101
Vombatus ursinus	Common wombat	XP_027715098
Zeugodacus cucurbitae	Melon fly	XP_011177346

[a]not applicable (*n.a.*)
[b]GenBank accession number
[c]Sequence of PARG1
[d]Sequence of PARG2

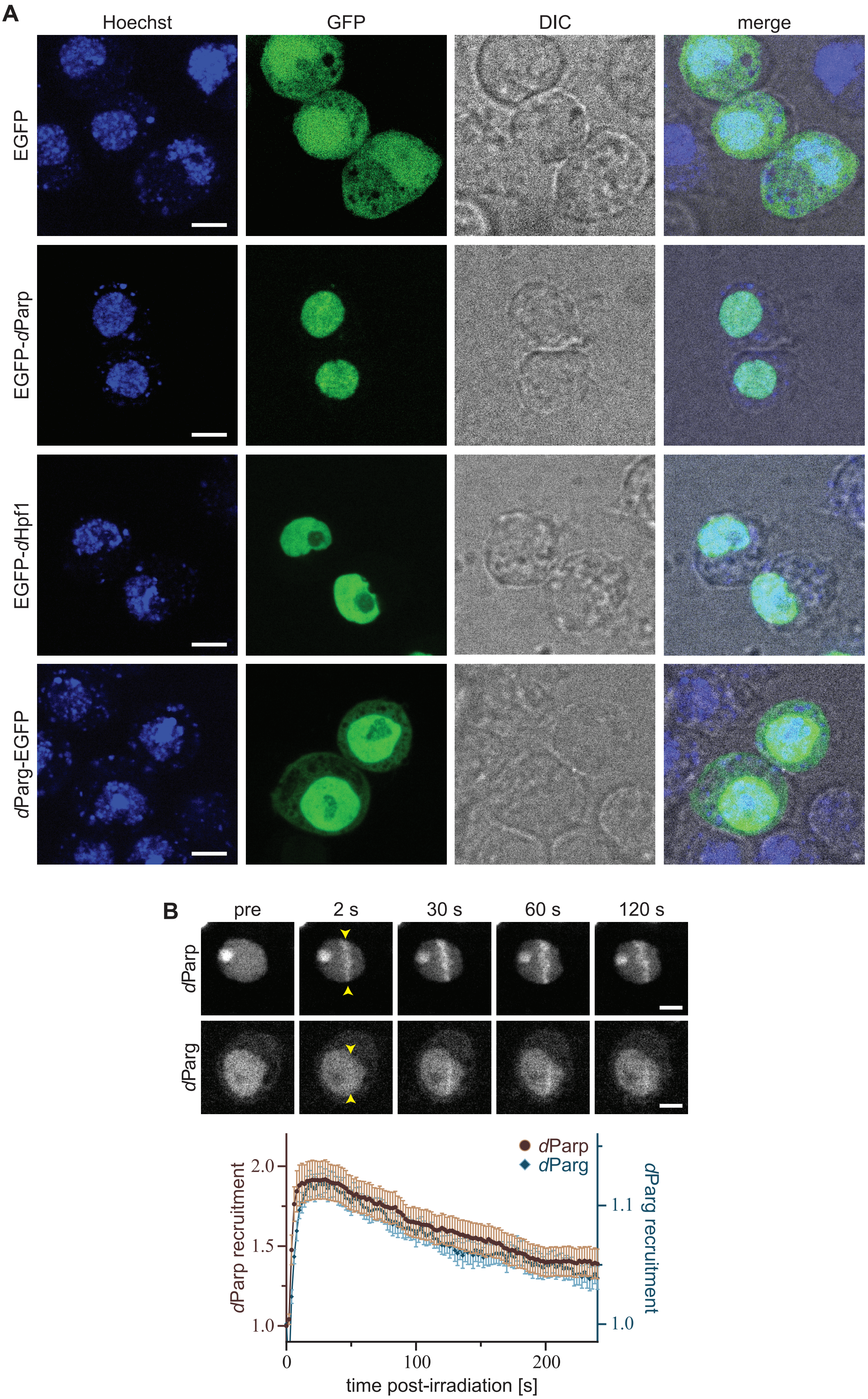
A
Hoechst
GFP
DIC
merge
EGFP
EGFP-dParp
EGFP-dHpf1
dParg-EGFP
B
pre
2 s
30 s
60 s
120 s
dParp
dParg
dParp
dParg
dParp recruitment
dParg recruitment
2.0
1.5
1.0
1.1
1.0
0
100
200
time post-irradiation [s]

Supplemental Figure 1. Subcellular localisation of the *Drosophila* DDR-ADPr enzymes.

(**A**) Representative images of EGFP, EGFP-*d*Parp, EGFP-*d*Hpf1 and *d*Parg-EGFP expressed in *Drosophila* SR2+ cells. Hoechst is used to indicate localisation of the nucleus. Scale bar, 2 µm. Sites of irradiation are indicated by yellow arrows. The experiment was repeated independently three times with similar results.

(**B**) Representative images (top) and kinetics (bottom) of EGFP-*d*Parp and *d*Parg-EGFP recruitment to sites of DNA damage induced by 405 nm laser irradiation, in *Drosophila* S2R+ cells. Scale bar, 2 µm. Data from B are a representative of 3 independent replicates where data were collected from 16-25 cells per condition and represent normalised mean values ± SEM.

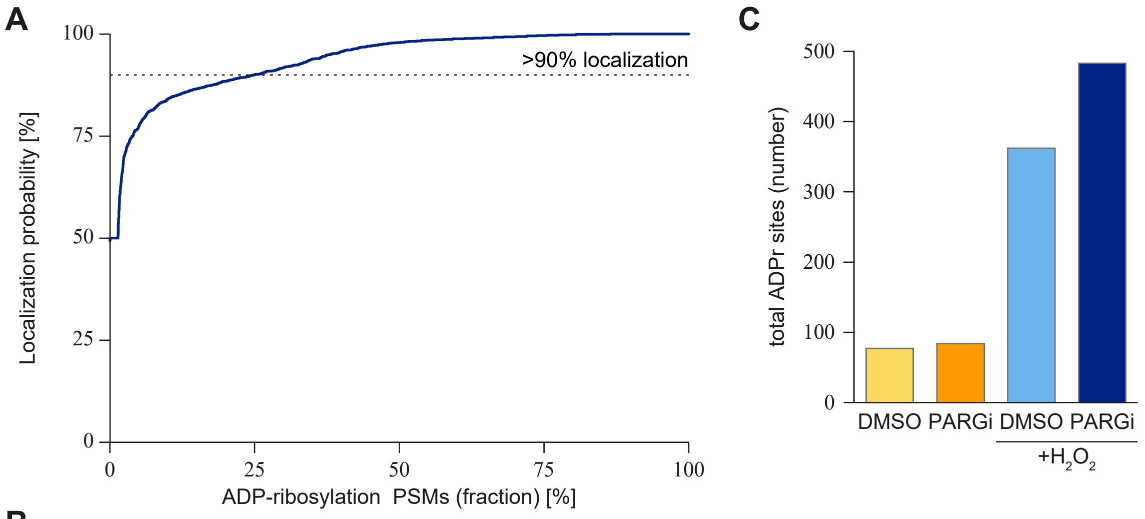

B

		DMSO + H_2O_2 R1	DMSO + H_2O_2 R2	DMSO + H_2O_2 R3	DMSO + H_2O_2 R4	DMSO R1	DMSO R2	DMSO R3	DMSO R4	PARGi + H_2O_2 R1	PARGi + H_2O_2 R2	PARGi + H_2O_2 R3	PARGi + H_2O_2 R4	PARGi R1	PARGi R2	PARGi R3	PARGi R4
DMSO + H_2O_2	R1		0.90	0.58	0.88	0.49	0.58	0.51	0.74	0.73	0.83	0.54	0.82	0.65	0.72	0.65	0.58
	R2	0.90		0.78	0.89	0.80	0.78	0.76	0.82	0.61	0.93	0.78	0.91	0.86	0.89	0.86	0.77
	R3	0.58	0.78		0.57	1.00	1.00	1.00	0.85	0.37	0.94	0.87	0.92	0.95	0.90	0.88	0.99
	R4	0.88	0.89	0.57		0.51	0.56	0.53	0.80	0.62	0.78	0.64	0.79	0.63	0.72	0.68	0.58
DMSO	R1	0.49	0.80	1.00	0.51		1.00	1.00	0.85	0.36	0.93	0.97	0.91	0.99	0.95	0.95	0.99
	R2	0.58	0.78	1.00	0.56	1.00		1.00	0.84	0.39	0.94	0.85	0.91	0.96	0.92	0.91	0.98
	R3	0.51	0.76	1.00	0.53	1.00	1.00		0.84	0.30	0.93	0.89	0.91	0.95	0.90	0.89	0.98
	R4	0.74	0.82	0.85	0.80	0.85	0.84	0.84		0.56	0.89	0.80	0.90	0.81	0.83	0.81	0.85
PARGi + H_2O_2	R1	0.73	0.61	0.37	0.62	0.36	0.39	0.30	0.56		0.58	0.29	0.60	0.38	0.47	0.39	0.37
	R2	0.83	0.93	0.94	0.78	0.93	0.94	0.93	0.89	0.58		0.85	0.99	0.93	0.93	0.89	0.93
	R3	0.54	0.78	0.87	0.64	0.97	0.85	0.89	0.80	0.29	0.85		0.82	0.87	0.86	0.86	0.86
	R4	0.82	0.91	0.92	0.79	0.91	0.91	0.91	0.90	0.60	0.99	0.82		0.88	0.88	0.85	0.92
PARGi	R1	0.65	0.86	0.95	0.63	0.99	0.96	0.95	0.81	0.38	0.93	0.87	0.88		0.99	0.96	0.94
	R2	0.72	0.89	0.90	0.72	0.95	0.92	0.90	0.83	0.47	0.93	0.86	0.88	0.99		0.98	0.91
	R3	0.65	0.86	0.88	0.68	0.95	0.91	0.89	0.81	0.39	0.89	0.86	0.85	0.96	0.98		0.91
	R4	0.58	0.77	0.99	0.58	0.99	0.98	0.98	0.85	0.37	0.93	0.86	0.92	0.94	0.91	0.91	

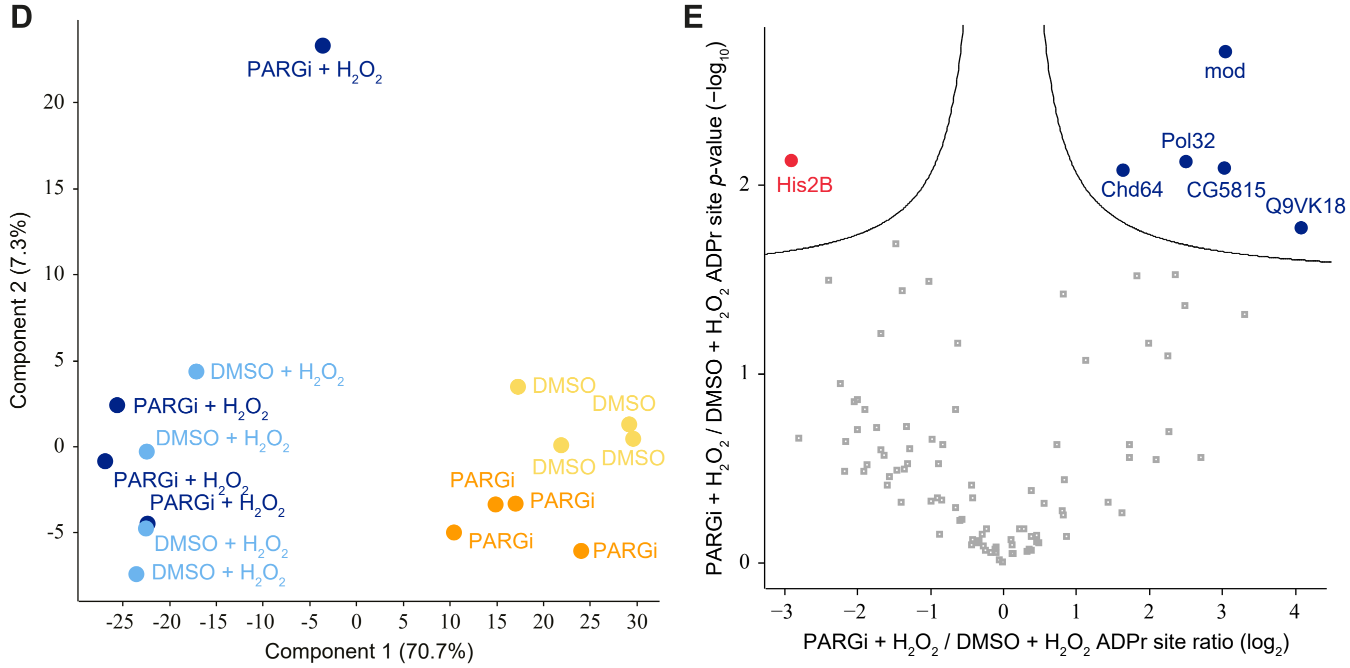

Supplemental Figure 2. Mass spectrometric identification of ADPr sites in S2R+ cells.

(**A**) Localization probability distribution of identified ADPr peptide-spectrum matches (PSMs). The dashed line visualizes the cutoff of >90% localization probability that was used.

(**B**) Table with all Pearson correlations between different replicates and different conditions.

(**C**) Histogram visualizing the total number of ADPr sites identified and localized in the four different conditions.

(**D**) Principal component analysis indicating the highest degree of variance between sample conditions.

(**E**) Volcano plot analysis visualizing the dynamics of PARGi-treated ADPr sites compared to DMSO-treated ADPr sites under DNA damage. Significance was determined via two-tailed Student's t-testing with a false discovery rate (FDR) of 0.05, s0 of 0.1, and 2,500 rounds of randomization. ADPr sites significantly upregulated by PARGi treatment are depicted in blue, ADPr sites significantly upregulated upon DMSO treatment are depicted in red, and ADPr sites not significantly regulated are shown in grey.

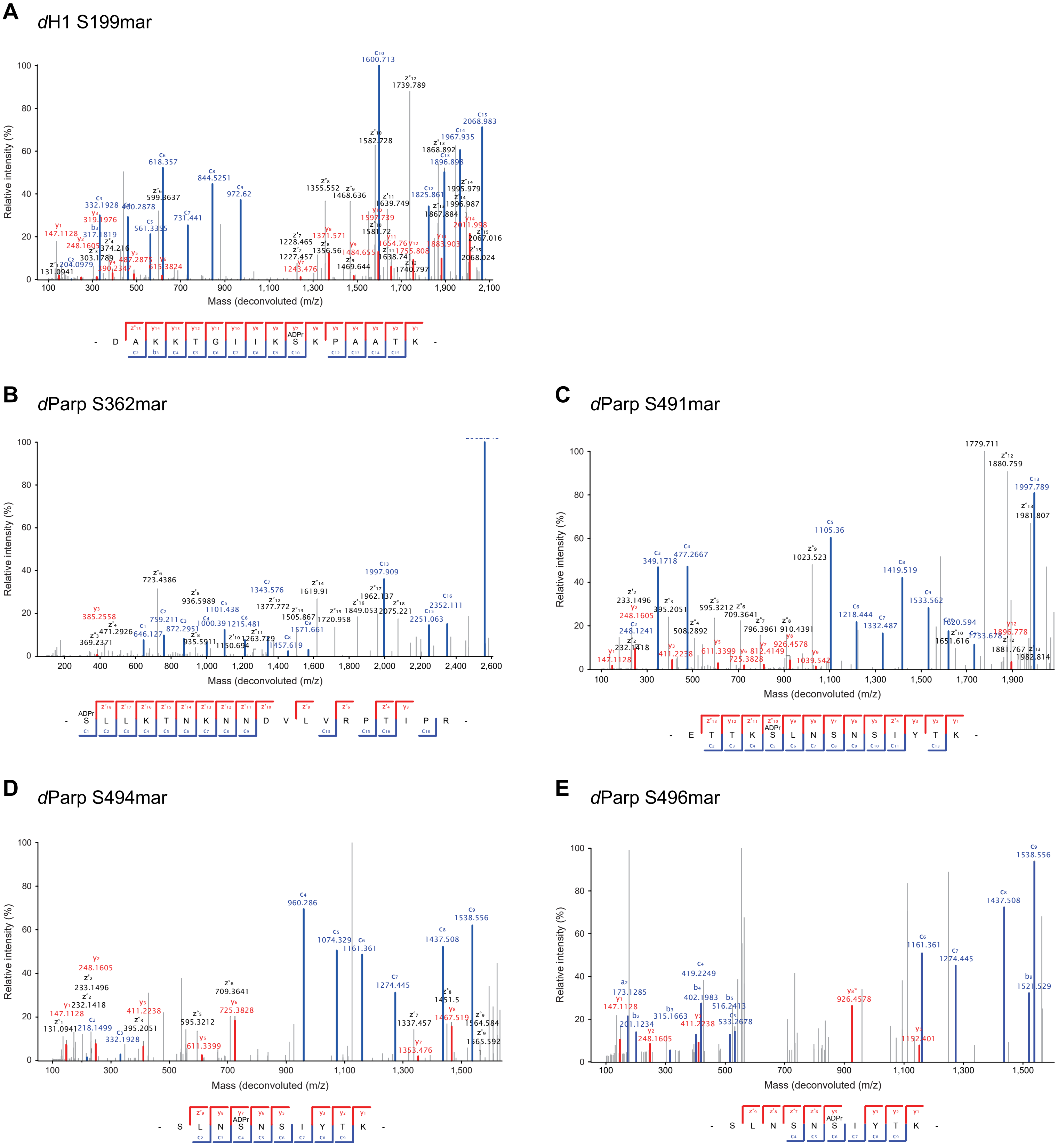
A
dH1 S199mar
Relative intensity (%)
Mass (deconvoluted (m/z)
- D A K K T G I I K S K P A A T K -
ADPr
B
dParp S362mar
Relative intensity (%)
Mass (deconvoluted (m/z)
- S L L K T N K N N D V L V R P T I P R -
ADPr
C
dParp S491mar
Relative intensity (%)
Mass (deconvoluted (m/z)
- E T T K S L N S N S I Y T K -
ADPr
D
dParp S494mar
Relative intensity (%)
Mass (deconvoluted (m/z)
- S L N S N S I Y T K -
ADPr
E
dParp S496mar
Relative intensity (%)
Mass (deconvoluted (m/z)
- S L N S N S I Y T K -
ADPr

Supplemental Figure 3. MS spectra of *Drosophila* histone H1 and *d*Parp Ser-ADPr sites.

(**A-E**) Annotated ETD tandem mass spectra of ADP-ribosylated peptides. Full fragmentation is obtained, with both c-ions (blue) and z-ions (red) pinpointing the ADPr to reside on a serine residue. The spectra allowed mapping of the peptides to *Drosophila* histone *d*H1 S199 (**A**), *d*Parp S363 (**B**), *d*Parp S491 (**C**), *d*Parp S494 (**D**), and *d*Parp S496 (**E**).

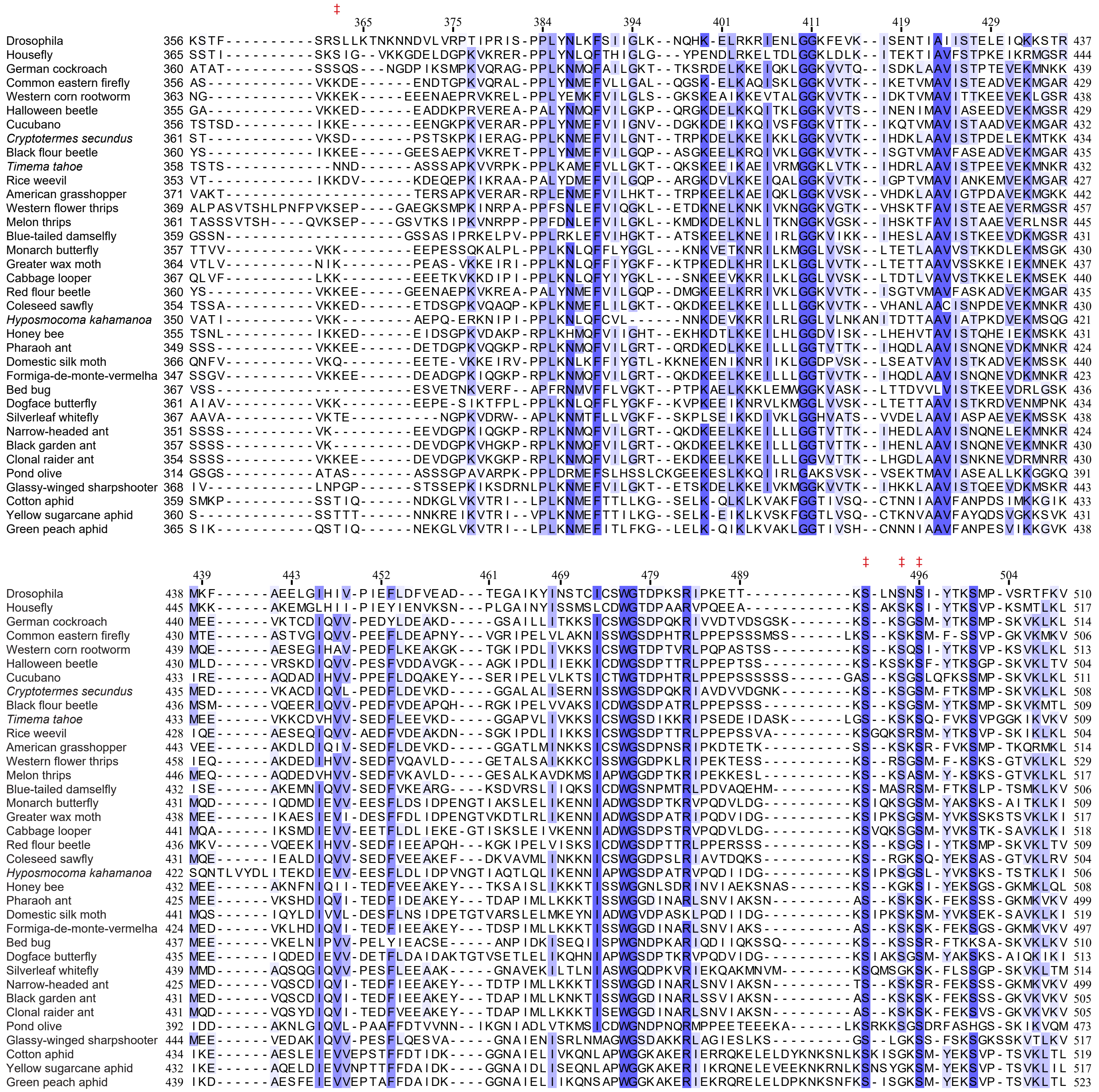

Supplemental Figure 4. Conservation of the PARP automodification domain in insects.

Multiple sequence alignment of selected insect PARP automodification domain sequences. Ser-ADPr sites identified in *d*Parp are indicated above the alignment by double-dagger (‡). Indexing indicates *d*Parp residue position.

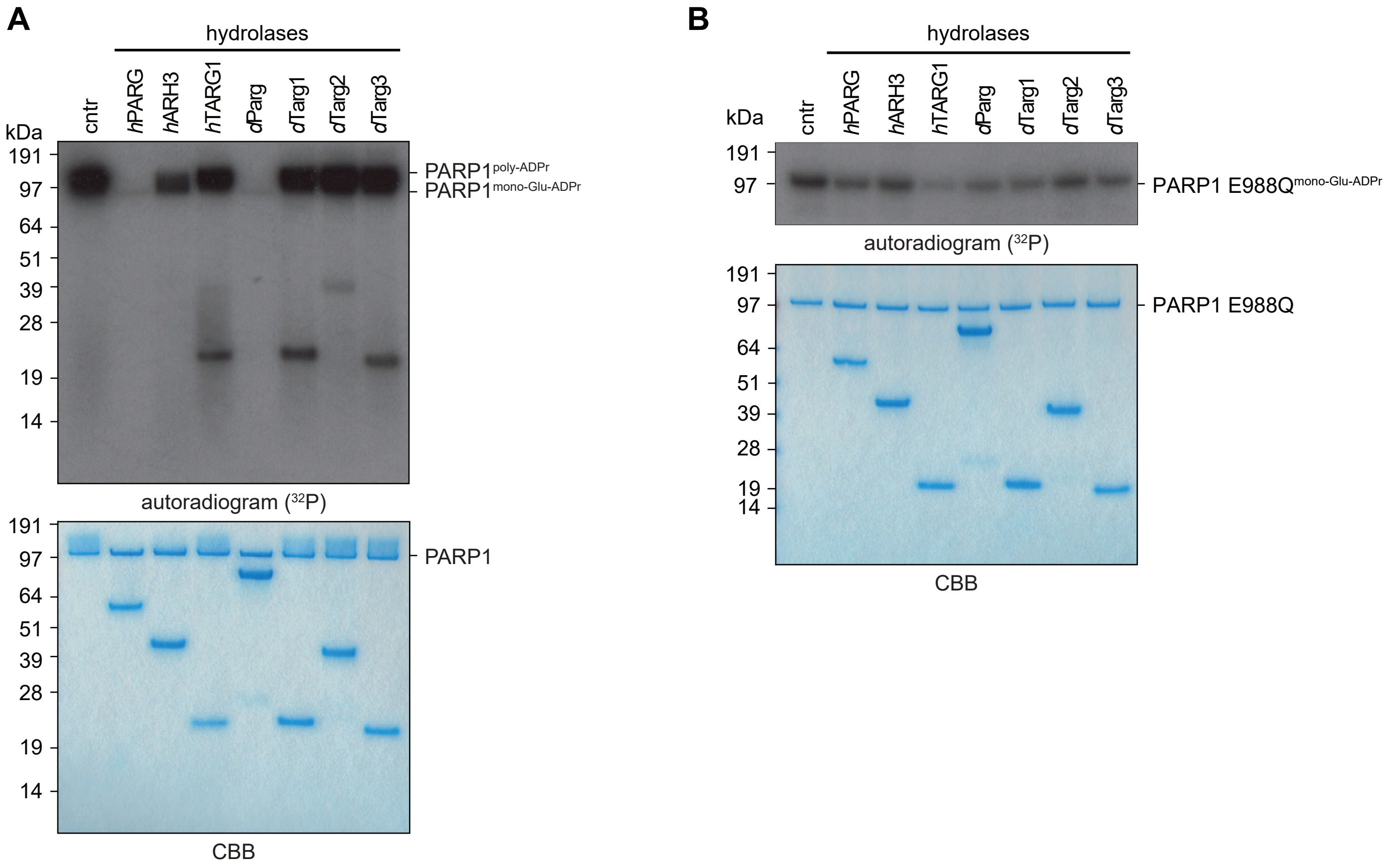
A
hydrolases
cntr
hPARG
hARH3
hTARG1
dParg
dTarg1
dTarg2
dTarg3
kDa
191
97
64
51
39
28
19
14
PARP1 poly-ADPr
PARP1 mono-Glu-ADPr
autoradiogram (32P)
PARP1
CBB
B
hydrolases
PARP1 E988Q mono-Glu-ADPr
PARP1 E988Q

Supplemental Figure 5. Residue specificity of *d*Parg terminal ADPr hydrolase activity.

(**A**) Removal of poly-Glu-ADPr by human and *Drosophila* (ADP-ribosyl)hydrolases on automodified poly-Glu-*h*PARP1. Poly-Glu-ADPr of *h*PARP1 was achieved by incubation of recombinant *h*PARP1 (0.5 μM) in the presence of ^{32}P-NAD^{+} as ADP-ribose donor. The reaction was performed for 30 min at room temperature. The *h*PARP1 reaction was stopped by the addition of 1 μM olaparib. Lower panel shows the CBB stained SDS-PAGE of the proteins. The experiment was repeated independently three times with similar results.

(**B**) Removal of mono-Glu-ADPr. Mono-Glu-ADPr was achieved by incubation of recombinant *h*PARP1 E988Q mutant (0.5 μM) with ^{32}P-NAD^{+} as ADP-ribose donor and activated DNA. The reaction of *h*PARP1was stopped by the addition of 1 μM olaparib and subsequently supplemented with the indicated (ADP-ribosyl)hydrolases. Lower panel shows the CBB stained SDS-PAGE. The experiment was repeated independently three times with similar results.

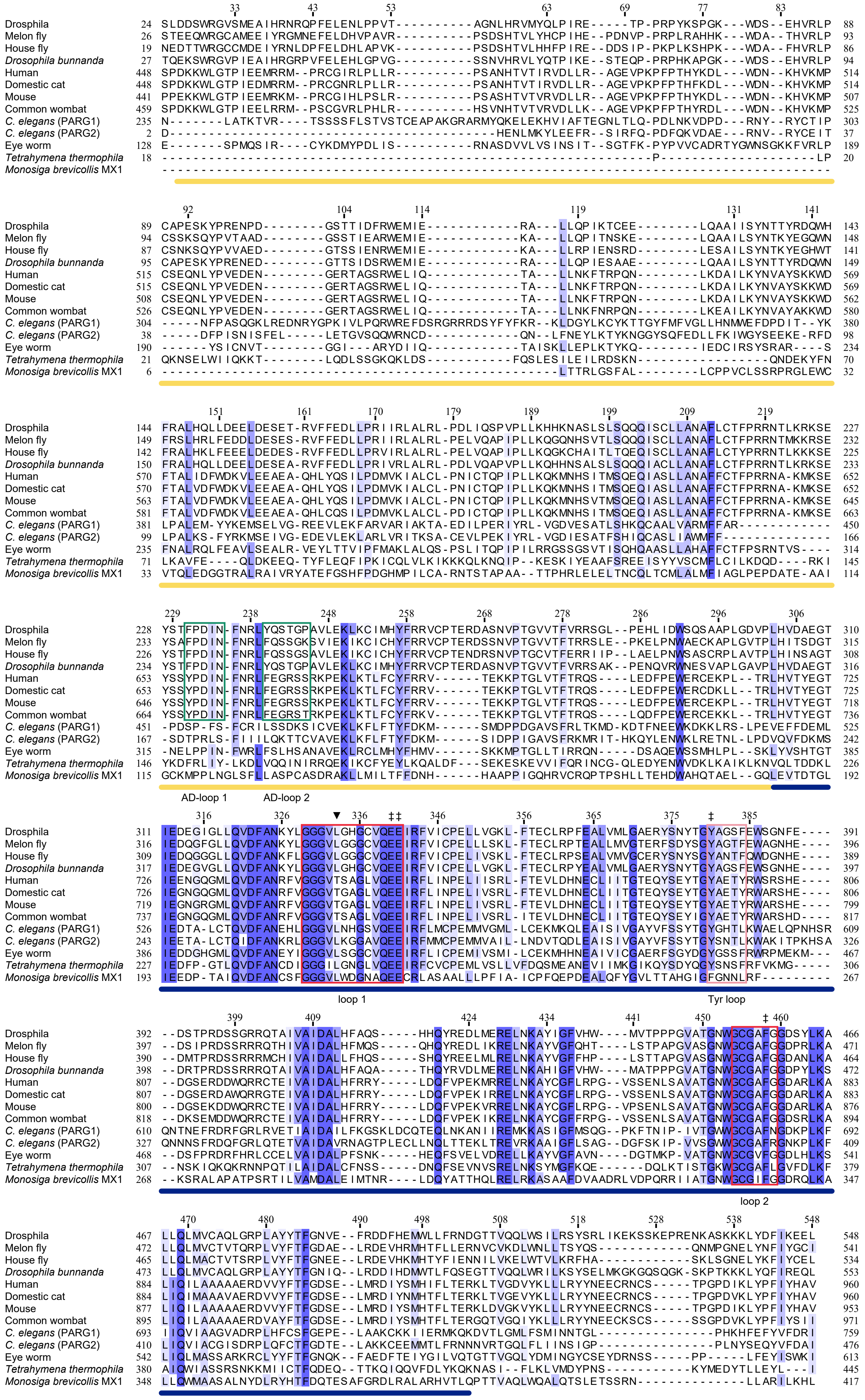
Drosphila
Melon fly
House fly
Drosophila bunnanda
Human
Domestic cat
Mouse
Common wombat
C. elegans (PARG1)
C. elegans (PARG2)
Eye worm
Tetrahymena thermophila
Monosiga brevicollis MX1
AD-loop 1
AD-loop 2
loop 1
Tyr loop
loop 2

Supplemental Figure 6. Conservation of PARG within *Animalia*.

Multiple sequence alignment of the catalytic domain of selected PARG sequences from *Insecta*, *Mammalia*, *Nematoda* and *Protozoa* phyla. The accessory (yellow) and macrodomain (blue) regions are indicated below the alignment and catalytically important regions highlighted by coloured boxes (AD-loop 1 and 2, green; loop 1 and 2, red; Tyr loop, rose). Residues known to be crucial for catalysis are indicated above the alignment by double-dagger (‡) and threonine/leucine residue involved in the loop 1-AD-loop 1 interaction by downward triangle (▼). Indexing indicates *d*Parp residue position.

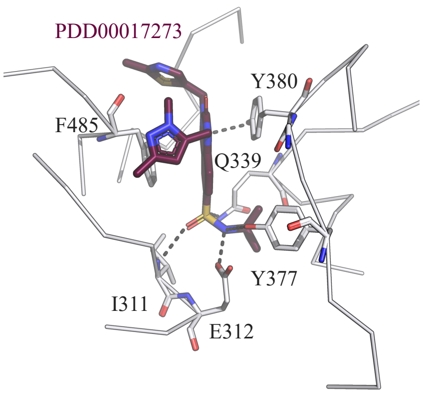

*d*Parg:PARGi (8ADJ)

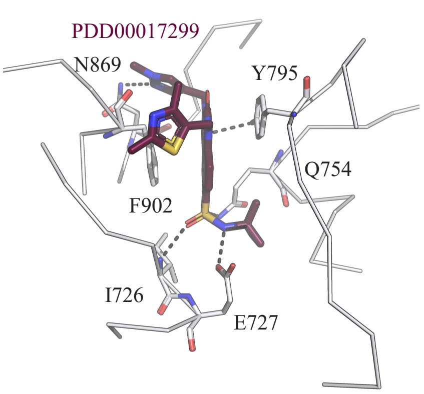

*h*PARG:PARGi (6HML)

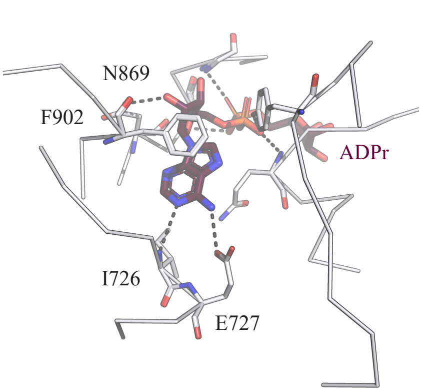

*h*PARG:ADPr (4B1H)

Supplemental Figure 7. Structure of the *d*Parg:PDD00017273 complex.

Ribbon-liquorice representation of the ligand coordination of the *d*Parg:PDD00017273, *h*PARG:PDD00017299, and *h*PARG:ADP-ribose complexes. Residues important for the protein:ligand interaction are highlighted and numbering corresponds to the respective sequences.

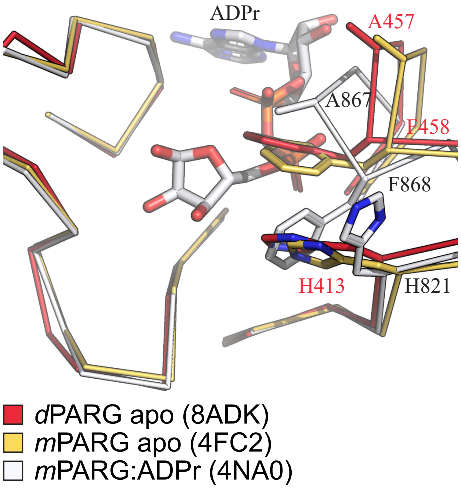
ADPr
A457
A867
F458
F868
H413
H821
dPARG apo (8ADK)
mPARG apo (4FC2)
mPARG:ADPr (4NA0)

Supplemental Figure 8. Ligand binding involves conformational changes of loop 2.

Ribbon-liquorice representation of loop 2 conformational changes triggered by ligand binding. Residue numbers of *d*Parg are given in red and of *m*PARG in black.
