# Supplementary material for: Serine ADP-ribosylation in Drosophila provides insights into the evolution of reversible ADP-ribosylation signalling
Source: Nat Commun. 2023 Jun 2;14:3200. doi: 10.1038/s41467-023-38793-y (PMC10238386; doi:10.1038/s41467-023-38793-y)
Supplement: Supplementary file 2 — Description of Additional Supplementary Files [file 41467_2023_38793_MOESM2_ESM.pdf]

Description of Additional Supplementary Files

File Name: Supplementary Data 1
Description: HPF1 sequences across the *Animalia* kingdom used for the phylogenetic analysis.

File Name: Supplementary Data 2
Description: Overview of ADPr sites identified using Af1521 enrichment followed by mass spectrometry.

File Name: Supplementary Data 3
Description: Oligonucleotides used in this study.
