# Supplementary material for: Serine ADP-ribosylation in Drosophila provides insights into the evolution of reversible ADP-ribosylation signalling
Source: Nat Commun. 2023 Jun 2;14:3200. doi: 10.1038/s41467-023-38793-y (PMC10238386; doi:10.1038/s41467-023-38793-y)
Supplement: Supplementary file 6 — Reporting Summary [file 41467_2023_38793_MOESM6_ESM.pdf]

nature portfolio

Corresponding author(s): Ivan Ahel
Michael L. Nielsen
Johannes G. M. Rack
Antonio Ariza

Last updated by author(s): Apr 20, 2023

Reporting Summary

Nature Portfolio wishes to improve the reproducibility of the work that we publish. This form provides structure for consistency and transparency in reporting. For further information on Nature Portfolio policies, see our Editorial Policies and the Editorial Policy Checklist.

Statistics

For all statistical analyses, confirm that the following items are present in the figure legend, table legend, main text, or Methods section.

n/a	Confirmed	
☐	☒	The exact sample size (*n*) for each experimental group/condition, given as a discrete number and unit of measurement
☐	☒	A statement on whether measurements were taken from distinct samples or whether the same sample was measured repeatedly
☐	☒	The statistical test(s) used AND whether they are one- or two-sided<br>*Only common tests should be described solely by name; describe more complex techniques in the Methods section.*
☐	☒	A description of all covariates tested
☐	☒	A description of any assumptions or corrections, such as tests of normality and adjustment for multiple comparisons
☐	☒	A full description of the statistical parameters including central tendency (e.g. means) or other basic estimates (e.g. regression coefficient) AND variation (e.g. standard deviation) or associated estimates of uncertainty (e.g. confidence intervals)
☐	☒	For null hypothesis testing, the test statistic (e.g. *F*, *t*, *r*) with confidence intervals, effect sizes, degrees of freedom and *P* value noted<br>*Give P values as exact values whenever suitable.*
☒	☐	For Bayesian analysis, information on the choice of priors and Markov chain Monte Carlo settings
☒	☐	For hierarchical and complex designs, identification of the appropriate level for tests and full reporting of outcomes
☐	☒	Estimates of effect sizes (e.g. Cohen's *d*, Pearson's *r*), indicating how they were calculated

Our web collection on statistics for biologists contains articles on many of the points above.

Software and code

Policy information about availability of computer code

Data collection	X-ray data acquisition: Generic Data Acquisition software (GDA, v.8.26, Diamond Light Source)<br>MS data acquisition: MaxQuant (v1.5.3.30) and Perseus (v1.5.5.3)
Data analysis	Publicly available software:<br>Statistical analysis and graph representation: Prism (v9.1, GraphPad)<br>Data reduction: Xia (v0.3.8)<br>Molecular replacement: Phaser (v2.7)<br>Density modification: PARROT (v0.8)<br>Automated model building: BUCCANEER (v1.5)<br>Data refinement: REFMAC5 (v5.0.32)<br>Manual model building: Coot (v0.9)<br>Software suit: CCP4i2 (v7), JalView (v2.11)<br>Structure validation: MolProbity (v4.5, Duke University)<br>Structure representation: PyMOL (v2.3, Schrödinger, LLC)<br>Multiple sequence alignment: MAFFT L-INS-i (v7.475)<br>Phylogenetic analysis: MEGA (v11)<br>MS data analysis: MaxQuant (v1.5.3.30), Perseus (v1.5.5.3), iceLogo (v1.2)

For manuscripts utilizing custom algorithms or software that are central to the research but not yet described in published literature, software must be made available to editors and reviewers. We strongly encourage code deposition in a community repository (e.g. GitHub). See the Nature Portfolio guidelines for submitting code & software for further information.

Data

Policy information about availability of data

All manuscripts must include a data availability statement. This statement should provide the following information, where applicable:

- Accession codes, unique identifiers, or web links for publicly available datasets
- A description of any restrictions on data availability
- For clinical datasets or third party data, please ensure that the statement adheres to our policy

The atomic coordinates included in the study have been deposited in the Protein Data Bank (PDB) with the following accession codes: apo dParg 8ADK, dParg:PARGi complex 8ADJ.
The mass spectrometry proteomics data have been deposited to the ProteomeXchange Consortium via the PRIDE partner repository with the dataset identifier PXD036512.
The imaging datasets generated and analysed during the current study are not publicly available as the large amount of imaging data could not be uploaded to a repository but are available from the corresponding author on reasonable request.
All other data generated in this study are provided in the Supplementary Information/Source Data file. Source data are provided with this paper.

Human research participants

Policy information about studies involving human research participants and Sex and Gender in Research.

Reporting on sex and gender	N/A
Population characteristics	N/A
Recruitment	N/A
Ethics oversight	N/A

Note that full information on the approval of the study protocol must also be provided in the manuscript.

Field-specific reporting

Please select the one below that is the best fit for your research. If you are not sure, read the appropriate sections before making your selection.

☒ Life sciences ☐ Behavioural & social sciences ☐ Ecological, evolutionary & environmental sciences

For a reference copy of the document with all sections, see nature.com/documents/nr-reporting-summary-flat.pdf

Life sciences study design

All studies must disclose on these points even when the disclosure is negative.

Sample size

Samples-size calculation was not performed. MS analyses for all primary experiments was performed in biological quadruplicate (independent cell cultures). The number of cells cultured for each replicate was based on previous reports and observations, in order to gain sufficient depth of sequencing and accurate profiling of low stoichiometry PTMs.

For crystallographic data, data were collected from single crystals and resolution limits were determined using data completeness and statistical parameters (incl. cc1/2, R values and I/sigma(I)).

For other experiments no statistical methods were used to determine sample size. For all experiments involving statistical analysis, experiments were performed in triplicates and as three independent biological replicas to ensure consistence within the experiments.

Data exclusions

No data were excluded.

Replication

Experiments were performed as at least three biological replicates. MS analyses was performed in biological quadruplicates. All attempts at reproduction were successful.

Randomization

Samples were not divided into experimental groups, all replicates for all individual experiments were simultaneously prepared, handled, and statistically processed while taking multiple-hypotheses testing into account.

Blinding

All samples relating to each experiment were handled simultaneously. During handling, all samples were numbered and processed in random order to avoid introduction of bias into the samples. During MS data acquisition, samples were clearly labeled (and thus not blinded), which is important to MS experimental design. The performance of the MS instrument drifts over time, and there can be power outages and other factors outside of our control. Therefore, it is important to run samples in an order where the least technical variance is introduced between runs (e.g. control_rep1, treatmentA_rep1, treamtentB_rep1, control_rep2, treatmentA_rep2, treatmentB_rep2, etc.). Further, this lets us account for the limited degree of sample carryover as a result from column carryover.

All data analysis was performed with unbiased software, in an unsupervised manner, and therefore blinding is not applicable in this context. All MS data is publicly available and may be re-processed and investigated by any external party.

Reporting for specific materials, systems and methods

We require information from authors about some types of materials, experimental systems and methods used in many studies. Here, indicate whether each material, system or method listed is relevant to your study. If you are not sure if a list item applies to your research, read the appropriate section before selecting a response.

Materials & experimental systems

n/a	Involved in the study
☐	☒ Antibodies
☐	☒ Eukaryotic cell lines
☒	☐ Palaeontology and archaeology
☒	☐ Animals and other organisms
☒	☐ Clinical data
☒	☐ Dual use research of concern

Methods

n/a	Involved in the study
☒	☐ ChIP-seq
☒	☐ Flow cytometry
☒	☐ MRI-based neuroimaging

Antibodies

Antibodies used

rabbit anti-poly ADPr antibody (cat# 4336-BPC-100, Trevigen, RRID: AB_2721257)
rabbit anti-pan ADPr reagent (cat# MABE1016, Millipore, RRID: AB_2665466)
anti-mono ADPr reagent (cat# MABE1076, Millipore, RRID: AB_2665469)
rabbit anti-poly ADPr anti reagent (cat# MABE1031, Millipore, RRID: AB_2665467)
rabbit anti-mono ADPr antibody (AbD33204, BioRad)
rabbit anti-phosphor Histone H2AvD (Ser137) antibody (600-401-914, Rockland, RRID: AB_828383)
mouse anti-phosphor Histone H2A.X (Ser139) antibody (clone JBW301, 05-636, Millipore, RRID: AB_309864)
mouse anti-actin monoclonal antibodies (clone JLA20, Developmental Studies Hybridoma Bank (DSHB), RRID: AB_528068)
polycolonal goat anti-mouse immunoglobulins/HRP (cat# P0447, Dako, RRID: AB_2617137)
polycolonal swine anti-rabbit immunoglobulins/HRP (P0399, Dako, RRID: AB_2617141)

Validation

The Mono-ADPr, PAN-ADPr and poly-ADPr binding reagents and anti-poly ADPr antibody were validated in this study using immunoblot to detect an increase in ADPr levels after H2O2 induced DNA damage.

Commercial antibodies were validated by the Manufacturer for their application in immunoblotting:
rabbit anti-poly ADPr antibody (cat# 4336-BPC-100, Trevigen, RRID: AB_2721257) https://www.bio-techne.com/p/antibodies/anti-par-polyclonal-antibody-rabbit_4336-bpc-100
rabbit anti-pan ADPr reagent (cat# MABE1016, Millipore, RRID: AB_2665466) https://www.merckmillipore.com/GB/en/product/Anti-pan-ADP-ribose-binding-reagent,MM_NF-MABE1016
rabbit anti-mono ADPr reagent (cat# MABE1076, Millipore, RRID: AB_2665469)
https://www.merckmillipore.com/GB/en/product/Anti-mono-ADP-ribose-binding-reagent,MM_NF-MABE1076
rabbit anti-poly ADPr anti reagent (cat# MABE1031, Millipore, RRID: AB_2665467)
https://www.merckmillipore.com/GB/en/product/Anti-poly-ADP-ribose-binding-reagent,MM_NF-MABE1031
rabbit anti-phosphor Histone H2AvD (Ser137) antibody (600-401-914, Rockland, RRID: AB_828383)
https://www.rockland.com/categories/primary-antibodies/histone-h2avd-phosphos137-antibody-600-401-914/
mouse anti-phosphor Histone H2A.X (Ser139) antibody (clone JBW301, 05-636, Millipore, RRID: AB_309864)
https://www.merckmillipore.com/GB/en/product/Anti-phospho-Histone-H2A.X-Ser139-Antibody-clone-JBW301,MM_NF-05-636-I
Mouse anti-actin monoclonal antibodies (clone JLA20, Developmental Studies hybridoma Bank (DSHB), RRID; AB_528068)
https://dshb.biology.uiowa.edu/JLA20
polycolonal goat anti-mouse immunoglobulins/HRP (cat# P0447, Dako, RRID: AB_2617137)
https://www.agilent.com/en/product/specific-proteins/elisa-kits-accessories/goat-anti-rabbit-immunoglobulins-hrp-affinity-isolated-2717113
polycolonal swine anti-rabbit immunoglobulins/HRP (P0399, Dako, RRID: AB_2617141)
https://www.agilent.com/en/product/specific-proteins/elisa-kits-accessories/swine-anti-rabbit-immunoglobulins-hrp-affinity-isolated-2717118

Eukaryotic cell lines

Policy information about cell lines and Sex and Gender in Research

Cell line source(s)	S2R+ (DGRC Stock 150 ; https://dgrc.bio.indiana.edu//stock/150 ; RRID:CVCL_Z831), U2OS (ATCC HTB-96, RRID: CVCL_0042)
Authentication	Cells were not routinely authenticated.
Mycoplasma contamination	Cells were routinely checked for mycoplasma contamination, and no contamination was detected.
Commonly misidentified lines (See ICLAC register)	No commonly misidentified cell lines were used in this study.
